# Current analysis of hypoxic-ischemic encephalopathy research issues and future treatment modalities

**DOI:** 10.3389/fnins.2023.1136500

**Published:** 2023-06-09

**Authors:** Hong-Qing She, Yi-Fei Sun, Li Chen, Qiu-Xia Xiao, Bo-Yan Luo, Hong-Su Zhou, Di Zhou, Quan-Yuan Chang, Liu-Lin Xiong

**Affiliations:** ^1^Department of Anesthesiology, Affiliated Hospital of Zunyi Medical University, Zunyi, China; ^2^Translational Neurology Laboratory, Affiliated Hospital of Zunyi Medical University, Zunyi, China; ^3^WANG TINGHUA Translation Institute, Affiliated Hospital of Zunyi Medical University, Zunyi, China; ^4^Institute of Neurological Disease, West China Hospital, Sichuan University, Chengdu, China; ^5^Department of Anesthesiology, Southwest Medical University, Luzhou, China

**Keywords:** hypoxic-ischemic encephalopathy (HIE), hypothermia, neural circuits, monkey, bibliometric analysis

## Abstract

Hypoxic-ischemic encephalopathy (HIE) is the leading cause of long-term neurological disability in neonates and adults. Through bibliometric analysis, we analyzed the current research on HIE in various countries, institutions, and authors. At the same time, we extensively summarized the animal HIE models and modeling methods. There are various opinions on the neuroprotective treatment of HIE, and the main therapy in clinical is therapeutic hypothermia, although its efficacy remains to be investigated. Therefore, in this study, we discussed the progress of neural circuits, injured brain tissue, and neural circuits-related technologies, providing new ideas for the treatment and prognosis management of HIE with the combination of neuroendocrine and neuroprotection.

## 1. Introduction

Hypoxic-ischemic encephalopathy (HIE) is a brain disorder resulting from inadequate blood and oxygen supply due to various causes. HIE most commonly occurs in neonates, also known as neonatal hypoxic-ischemic encephalopathy (NHIE), with perinatal asphyxia as the main cause. During the perinatal period, umbilical cord entanglement or abnormal amniotic fluid can cause fetal distress, asphyxia, and hypoxia. Non-NHIE can be seen in severe cerebral hypoxia-ischemia (HI) from a variety of causes, including shock, respiratory and cardiac arrest, carbon monoxide poisoning, myasthenia gravis, and persistent status of epilepticus. Meanwhile, tissue damage has been observed in the cerebral cortex, hippocampus (Hipp), striatum (STR), and thalamus (TH), as well as the subcortical and periventricular white matter injury (WMI) has been found (Vannucci and Vannucci, 2005).

Bibliometric analysis is a mathematical statistical-based method that allows qualitative and quantitative analyses of contributions and collaborations in different countries or regions, institutions, authors, and journals. To date, bibliometric analysis has been performed in a wide range of medical fields with many overviews of scientific productivity, such as stem cell extracellular vesicles and esketamine (Li et al., 2022; Qiu et al., 2022). There is substantial evidence that every leap in understanding basic research into the etiology and pathogenesis of disease has been accompanied by an enrichment of therapeutic approaches and technological advances, which in turn have been expanded in subsequent clinical practice, leading to a marked improvement in treatment. The choice of experimental animals is the primary consideration in scientific research. In this article, we listed the advantages of various animals in HIE research. In general, rodent (mouse and rat) models can be more cost-effective and offer greater flexibility (Landucci et al., 2022), however, there are significant differences in brain structure and maturation between rodents and humans, which greatly hinder the full translational applicability of rodent findings to humans. Moreover, severe surgical trauma and long recovery time restrict the use of pregnant sheep as a model for HIE studies. Non-human primates are believed to be the most ideal and appropriate animal models for HIE studies because they are the most evolved and closest to humans, such as the macaques (Kobayashi et al., 2018; Guan et al., 2023). The acute perinatal asphyxia model has been successfully established in non-human primates using umbilical cord obstruction (UCO) to induce moderate to severe HIE before delivery (Traudt et al., 2013; McAdams et al., 2017). In the presence of severe asphyxia, the animals developed cerebral palsy-like motor abnormalities (Traudt et al., 2013; McAdams et al., 2017). Besides, the stroke model can be induced by stereotaxically injecting the autologous anticoagulants into the macaque’s brain (Zhu et al., 2011).

After years of clinical practice, a growing number of clinical treatments for HIE are available worldwide, including, but not limited to, hypothermia, xenon therapy, erythropoietin therapy (Lepore and Fischer, 2005), hyperbaric oxygen therapy, and melatonin therapy. Despite increasing clinical evidence that these therapies exert protective effects on HIE, the accompanying lifelong neurodevelopmental deficits, including cerebral palsy, impaired intelligence, and Intellectual disability cannot be resolved satisfyingly. In animal models, decreased body temperature during neonatal asphyxia has been demonstrated to increase cerebral antioxidant capacity. However, in preterm or severely asphyxiated neonates, instead of being beneficial, this therapy seems to be harmful (Kletkiewicz et al., 2020). Therefore, it is crucial to seek new therapeutic approaches to prevent anoxia-caused complications.

Perhaps studies of neural circuits could help us find new treatments. Neural circuits provide the anatomical basis for functional networks, complex and precise networks that control different functions such as movement, sensation, cognition, emotion, learning, and memory through the interconnection of neurons. Dissecting the structure of neural circuits is therefore essential to understanding how the brain works. It has been shown that neural circuits can regulate the immune response by detecting inflammatory mediators and feeding signals back to the immune system (Chavan and Tracey, 2014). Specific neuroendocrine patterns can restore nerve recovery and be used in the treatment of Alzheimer’s disease (Chan et al., 2021). The emerging neuromodulation techniques may provide effective strategies to alleviate HIE by assisting traditional treatments (therapeutic hypothermia and pharmacotherapy). Electroacupuncture has been proven to have antidepressant effects by repairing neural circuits and promoting synaptic plasticity (Feng et al., 2022). Acupuncture can delay the progression of Parkinson’s disease by regulating the balance of dopaminergic circuits and reducing the neurodegeneration of dopaminergic neurons (Zhao et al., 2021). Well understanding the plasticity of neural circuits may contribute to finding new therapies for these diseases. It has been found that in higher mammals, neural circuits can be partially reconstructed and functionally restored using exogenously transplanted neural stem cells or endogenous neurons, while some lower vertebrates can regenerate through spontaneous neurogenesis after neurological injury (Peng et al., 2022). It is believed that with the development of neuroscience and ongoing research on the pathogenesis of HIE, further progress will be made in the treatment of HIE.

At present, the current approach to the care of newborns with HIE does not result in a satisfactory prognosis for patients. Although they have certain antioxidant and protective effects on brain tissues, the concurrent problems of cerebral palsy, intellectual impairment, and Intellectual disability remain to be addressed. Taking this into account, in this review, we repeatedly studied animal HIE models and comprehensively summarized the modeling methods and selection of HIE models. Meanwhile, the elements and formation mechanisms of mammalian neural circuits, as well as the methods and cases of regulating neural circuits, are reviewed to provide new therapeutic strategies for HIE to enhance neuroprotection and/or neurodegeneration and to lay the foundation for possible treatment of HIE by studying neural circuits in the future.

## 2. Bibliometric analysis

**Figure 1:**
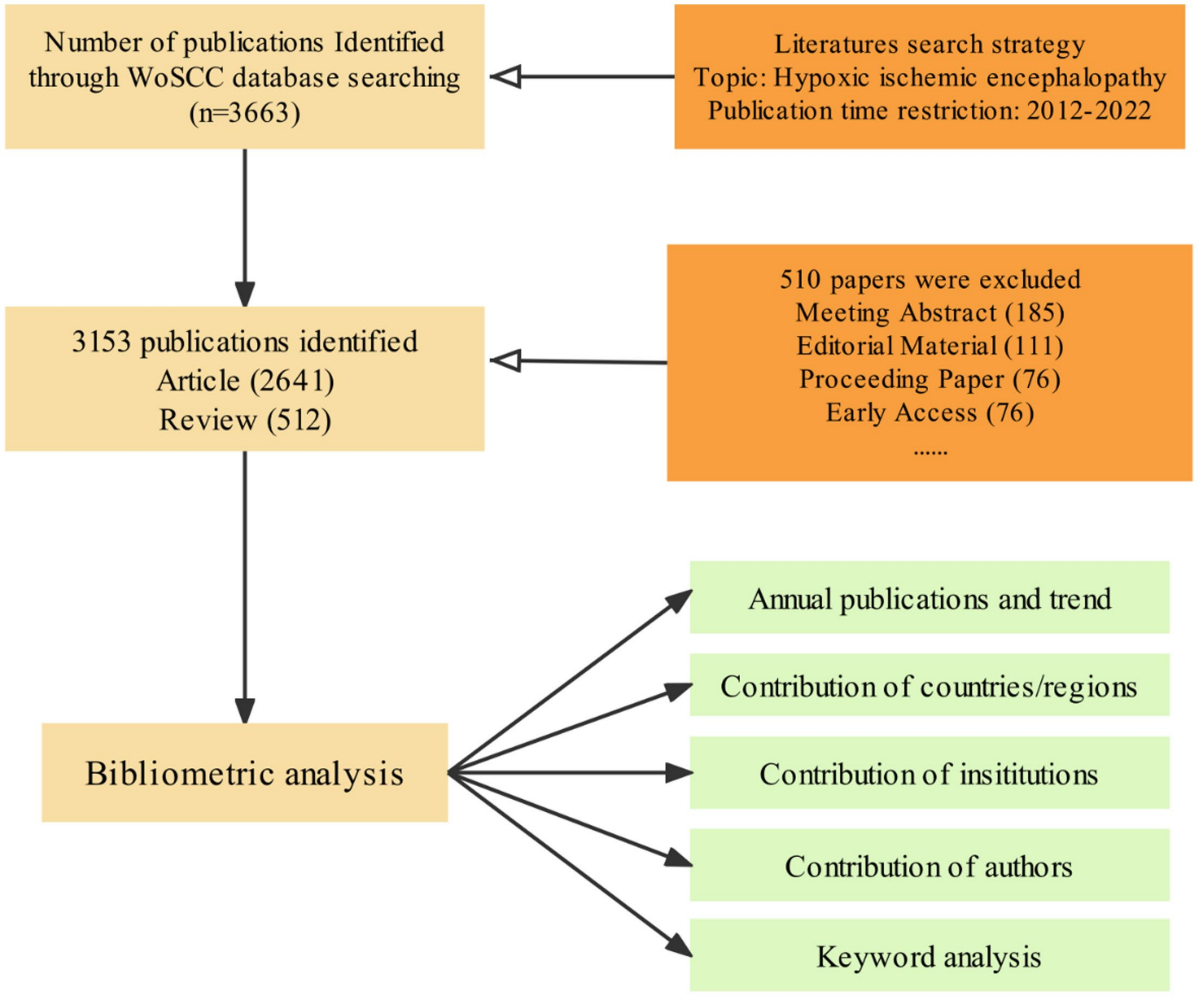
Flow chart of the screening process for research on the HIE.

The HIE-related publications from 2012 to 2022 years were searched by the WoSCC database on October 9, 2022, and the retrieval strategy and filtering process was shown as shown in Figure 1. All publications that met the retrieval strategy were reviewed, except for the conference abstract, editorial material, proceedings, etc. The research trends and hotspots were analyzed using VOSviewer, Scimago Graphica, and CiteSpace.

### 2.1. Annual publications and trends

**Figure 2:**
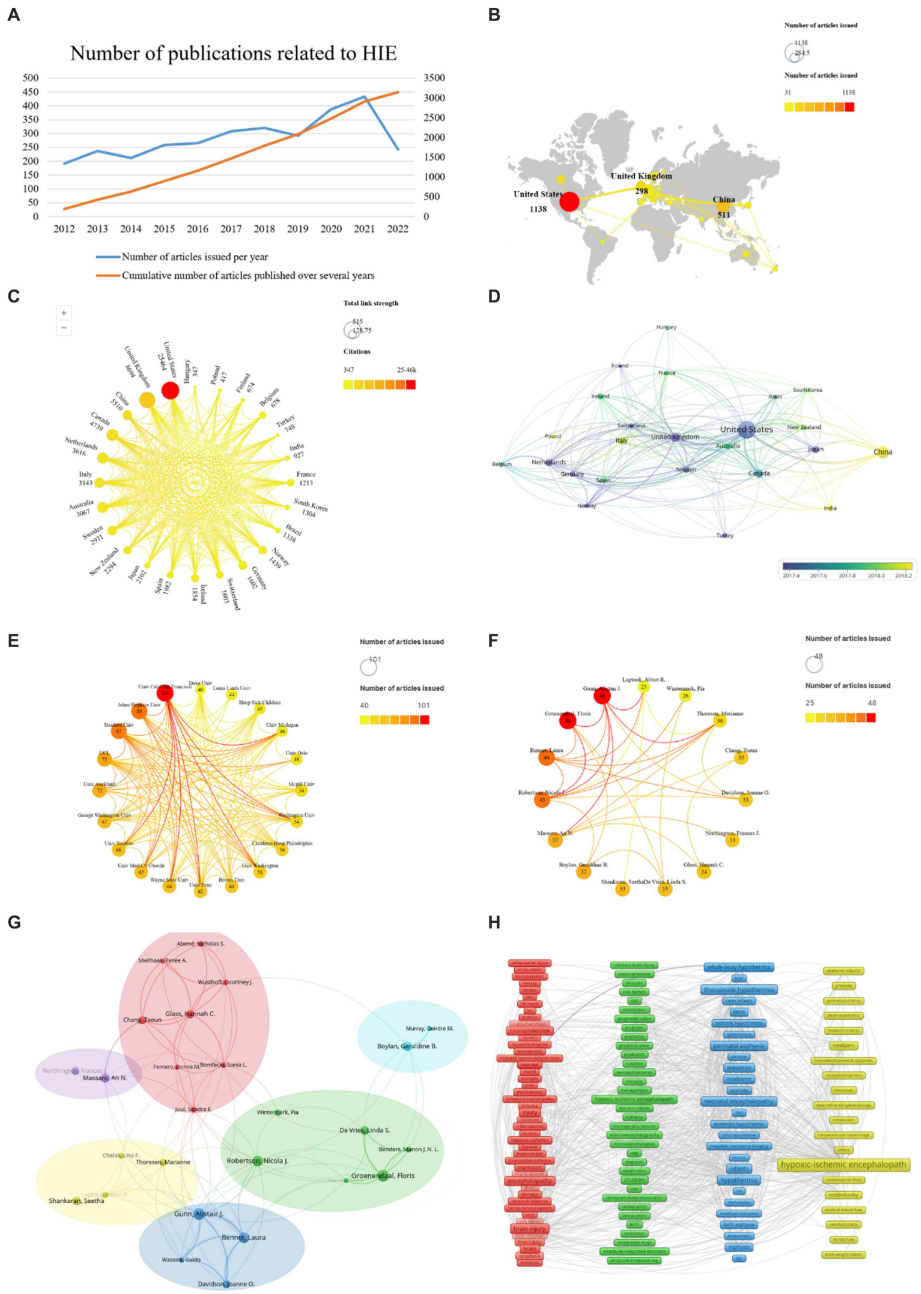
HIE-related studies published worldwide. **(A)** Annual and cumulative number of HIE-related papers published worldwide from 2012 to 2022. **(B)** Network of the country distribution and the total number of HIE-related papers published worldwide. **(C)** Graph of total citations and total link strength (TLS) of HIE-related publications worldwide. **(D)** Chronological order of countries. **(E)** Collaboration between the institutions. **(F)** Collaboration between authors. **(G)** Analysis of collaborative relationships between authors who have published at least 20 papers on the HIE. Each node represents an author, and the size of the node indicates the number of publications. The connections between nodes represent co-authorship relationships, and the thickness of the line indicates the strength (weight on the TLS). **(H)** Clustering analysis of keywords.

A total of 3,663 HIE-related publications were obtained from the WoSCC database encompassing the past 10 years. Of these 3,153 eligible publications, “Article” was the predominant publication type (83.76%, 2,641/3,153, Figure 1). The trend in HIE research has been steadily increasing. Despite a slight decline in 2019, the number of publications in the HIE field continues to trend upward annually (Figure 2A).

### 2.2. Countries or regions

Ninety-one total countries/regions have published HIE-related studies. The total number of papers published all over the world so far is 3,153, and it is increasing every year (Figure 2A). We analyzed those 24 countries that published more than 30 publications (Figure 2B). It is worth noting that the United States was the top publisher with 1,138 papers, followed by China with 511 papers, followed by the United Kingdom with 298 papers. In terms of total citations, the top three countries were the United States (25,464 citations), the United Kingdom (8,694 citations) and China (5,510 citations). The countries with the highest TLS were as follows: the United States (515), the United Kingdom (420) and China (150) (Figure 2C) indicating the dominant influence of the United States in this field, followed by the United Kingdom and China. As shown in Figure 2D, the average year of publication for papers in China was 2018, while the average year of publication for papers in the United States and the United Kingdom was around 2017.

### 2.3. Institutions

A total of 3,198 different institutions were involved in the publication of HIE-related studies, of which 20 institutions published at least 40 papers. Among them, University of California, San Francisco was the highest publisher with 101 papers, followed by Johns Hopkins University and Stanford University with 89 and 87 papers on HIE field, respectively (Figure 2E).

### 2.4. Authors

A total of 13,411 authors contributed to the research on HIE, and 15 authors published at least 25 papers. Groenendaal, Floris, Gunn, and Alistair J. were the most productive and insightful authors in the field of HIE, with 48 papers (Figure 2F). Besides, authors who published at least 20 articles were included in the author network map and divided into six clusters. Gunn, Alistair J. (TLS = 109), Glass, Hannah C. (TLS = 76), Groenendaal, Floris (TLS = 61), Boylan, Geraldine B. (TLS = 25), Massaro, An N. (TLS = 25), and Shankaran, Seetha (TLS = 19) were located at the center of these clusters, respectively (Figure 2G).

### 2.5. Keywords analysis

To track the trends and hot topics in the field of HIE, we performed a keyword co-occurrence analysis using the VOS viewer. The results showed there were 9,210 keywords available in the 3,153 publications, and the top 20 most frequent keywords were listed in Table 1. As shown, “hypoxic-ischemic encephalopathy” was the most frequent keyword (1,354 co-occurrences), which was consistent with our research topic. As represented in the co-occurrence network (Figure 2H), the identified keywords of HIE can be divided into four clusters as follows: pathophysiological mechanisms (red zone), prognosis (green zone), current mainstream treatments (blue zone), and populations (yellow zone). Notably, “hypothermia” was the second most frequent keyword in the list with 566 occurrences, and newborns and preterm infants were the main populations affected by HIE.

**Table 1:** Top 20 keywords in occurrence frequency.

Rank	Keywords	Occurrences	Total link strength
1	Hypoxic-ischemic encephalopathy	1354	11369
2	Hypothermia	566	4891
3	Therapeutic hypothermia	565	5088
4	Brain-injury	493	4437
5	Perinatal asphyxia	474	4404
6	Neonatal encephalopathy	457	4207
7	Infants	442	3732
8	Encephalopathy	392	3237
9	Neuroprotection	343	3215
10	Whole-body hypothermia	338	3123
11	Injury	320	2636
12	Outcomes	297	2547
13	Newborns	281	2526
14	Brain	268	2291
15	Asphyxia	267	2372
16	Newborn	242	2122
17	Hypoxic ischemic encephalopathy	226	1723
18	Term	210	1747
19	Children	208	1547
20	Hypoxia-ischemia	180	1688

### 2.6. Conclusion

In general, the HIE field is still in a rapid development stage and the research area is expanding. There is no doubt that the United States is at the forefront of global research, followed by China and the United Kingdom. In terms of citations, the total number of citations and TLS in China is low compared to the United Kingdom, but the number of published papers is higher, suggesting the papers published in China maybe has a small impact. It was concluded that the current mainstream therapeutic approach for HIE is still hypothermia, according to the keyword analysis. However, the effectiveness of this treatment is limited and about half of the children with moderate to severe HIE, even after hypothermia treatment, still suffer from considerable complications, such as moderate to severe disability or even the threat of death (Victor et al., 2022). Therefore, there is an urgent need to investigate a novel treatment method that can effectively protect the neurology of children with HIE and reduce serious sequelae. For a better study of HIE disease, more optimized animal models should also be used.

## 3. Experimental models for cerebral ischemia

### 3.1. Modeling methods

**Figure 3:**
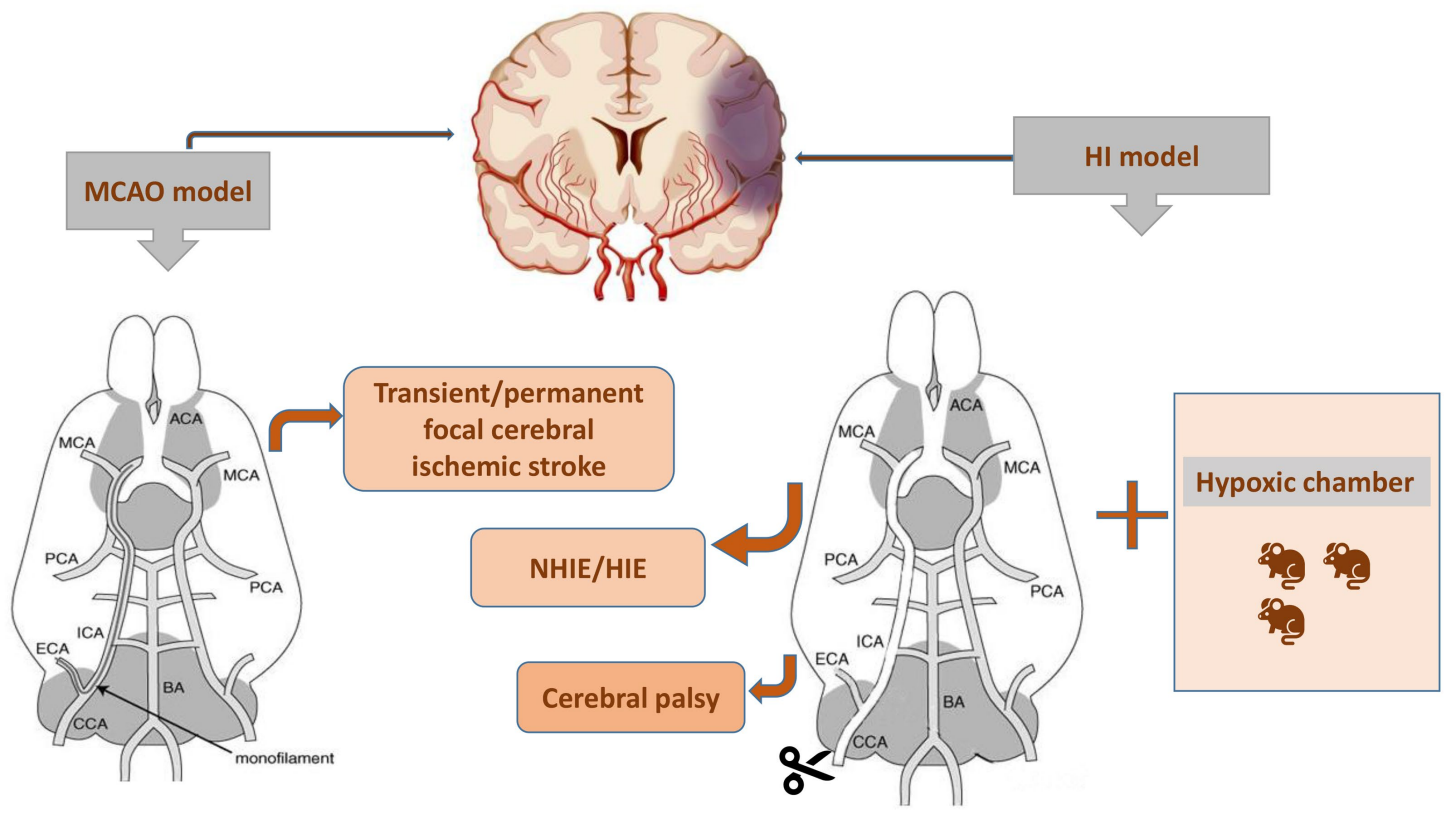
Two procedures for establishing the animal models with brain diseases. MCAO, middle cerebral artery occlusion; NHIE, neonatal hypoxic-ischemic encephalopathy; HIE, hypoxic-ischemic encephalopathy; HI, hypoxia-ischemia.

Inducing controlled and consistent injury in experimental animals allows us to investigate the cellular and molecular mechanisms of the brain after injury and explore novel potential therapeutic medicines. HI-induced cerebral edema and infarction along with long-term irreversible neurodevelopmental disorders, such as ischemic stroke and cerebral palsy, all resulted from inadequate blood and oxygen delivery to the brain. Researchers are prone to establishing HI models by disrupting the critical routes of blood delivery to the brain. Focal ischemia is induced by transient or permanent occlusions and is commonly used in basic research to imitate stroke conditions in humans. Conventionally, thread embolism has been used for transient focal ischemia and reperfusion (Durukan and Tatlisumak, 2007). Since 1981, the method of middle cerebral artery (MCA) occlusion (MCAO)-induced focal cerebral ischemic stroke in rodents has been generally used (Tamura et al., 1981). For analysis of reperfusion-mediated brain injury, a filament is inserted into the external carotid artery (ECA) and reperfusion occurs upon withdrawing the filament in a transient MCAO model (Longa et al., 1989). In 1981, Vannucci et al. first proposed a method for establishing an HIE model (Rice et al., 1981), which involved two steps: ligation of the right common carotid artery (CCA) followed by exposure to hypoxia (Figure 3). Cerebral blood flow in the ipsilateral hemisphere of the ligated CCA is not reduced by collateral blood flow via the circle of Willis (cerebral arterial circle), but it is significantly reduced in the ipsilateral hemisphere as oxygen tension decreases, leading to unilateral ischemic injury (Taniguchi and Andreasson, 2008). This method covers the shortcomings of other modeling methods and improves the efficiency of HI modeling. In addition to the Rice-Vannucci model, there are purely hypoxic, inflammation-induced perinatal brain injury and alternative models, such as immersion of the uterine horns containing the fetuses in a water bath during cesarean section in rats at embryonic day 21/22, transient umbilical cord occlusion in ovine fetuses to induce global HI, and intrauterine occlusion of the descending aorta to induce HI in preterm rabbit fetuses (Millar et al., 2017). Nevertheless, the Rice-Vannucci model of neonatal HI is the most widely used (Yager and Ashwal, 2009). Rodents on postnatal day 7 (P7), especially the rats and mice, are usually chosen for HI modeling because the brain growth of these animals reaches a peak at this age. Besides, P7 rodents have just started cortical myelination, similar to a human fetus at 34 weeks of gestation (Bennet et al., 2012). This model simulating neonatal HI is supported by a series of behavioral evaluations, such as the Zea-Longa score, neurologic severity score, Morris water maze test, Y maze test, rotarod test, and negative geotaxis test, to detect their neurological functions (Arteni et al., 2003). Additionally, cerebral palsy as a neurodevelopmental disorder is usually attributed to HI in perinatal neonates (Vitrikas et al., 2020), and therefore the models of cerebral palsy in existing studies were established in neonates subjected to the HI methods (Wen et al., 2020).

### 3.2. Criteria for animal selection

Besides the technique used to occlude the blood vessels, the reliability of animal models heavily depends on many factors, for example, the strain or age of animals. The ideal cerebral ischemia model should meet the following criteria: (1) the process and the pathophysiological response of ischemia are similar to that of human cerebrovascular diseases; (2) the size of the ischemic lesions is highly reproducible; (3) the modeling techniques are relatively easy and less invasive; (4) x physiological variables can be monitored and maintained within the normal range; (5) brain samples are easily available for histological, biochemical, and molecular biology studies; and (6) animals are cost-efficient (Hermann et al., 2019). Also, some researchers have emphasized that the establishment of reliable rat models requires the selection of animals suitable for stable and long-term experiments regarding the mortality, cerebral blood flow, and extension of ischemic lesions induced by ischemic injury (Taniguchi and Andreasson, 2008).

### 3.3. Types of experimental animals for HI

Currently, scholars at home and abroad apply a variety of animals to establish cerebrovascular disease models, such as cats, dogs, rabbits, pigs, primates, rats, and mice (Table 2).

**Table 2:** Selection of animals for HIE-related studies.

Animals	HI model	Advantages
Rats	+	Little cerebrovascular variation; cost-efficient; easy access and caring conditions (Svoboda et al., 2019).
Cats	+	Distribution pattern of blood vessels close to humans (Alger et al., 1989).
Rabbits	+	Ear veins provide convenience for injection administration and blood collection (Kumazawa et al., 2008).
Sheep	+	Better comparison of brain development at birth (Dean et al., 2015).
Piglets	+	Better comparison of brain development at birth (Roohey et al., 1997).
Dogs	+	Better neurological and circulatory systems (Zwemer et al., 1995).
Monkeys	+	Well-developed central nervous system; shorter reproduction cycle; extremely similar reactivity to drugs, functions, metabolism, blood biochemical characteristics, and cranial and brain development to humans (Kobayashi et al., 2018).

+, yes.

Rodents are most commonly used for investigating cerebral infarction, among which rats are the most prominent because their nervous system is similar to that of humans with little cerebrovascular variability, and their use is relatively cost-effective due to easy access and care conditions. Some data indicate that high variability among the rat strains might negatively influence stroke induction by intraluminal thread occlusion in the MCA (Svoboda et al., 2019). Nowadays, of the three widely used outbred rat strains, Sprague–Dawley rats are the most trustworthy outbred rat strain in the ischemic model on account of the maximal reduction of cerebral blood flow and extensive ischemic lesions in these rats. Comparatively, higher mortality is exhibited in Wistar rats, and significantly smaller or no ischemic region is found in Long-Evans rats. Although non-rodents are quite representative of neonatal HI to simulate the clinical symptoms of humans, a minority of researchers have investigated larger animals such as pigs, sheep, and macaques (Roohey et al., 1997; Dean et al., 2015).

Large animals own the advantages of replicating the conditions of a single human fetus exposed to a non-sterile environment, allowing better comparison of brain development at birth. Currently, a preclinical animal model of neonatal hypoxic-ischemic brain injury is established in ovine fetuses, which are subjected to global HI through transient UCO (Ophelders et al., 2016). To evaluate the safety and potential neuroprotective effects of some medicines for HI treatment, piglets have also been applied. Besides, it was revealed that high-doses of cannabidiol could induce severe hypotension and did not provide neuroprotection in the piglets at the early stage after global HI (Garberg et al., 2017).

Due to dogs have better neurological and circulatory systems and their responsiveness to drugs is close to that of humans, making it easy to establish conditioned reflexes for long-term experimental research, which makes them suitable for microsurgical studies of cerebrovascular disease (Zwemer et al., 1995). Similarly, the distribution of blood vessels in cats is close to that of humans and therefore is also commonly used in the establishment of cerebral infarction models (Alger et al., 1989). The ear veins of rabbits provide convenience for injection administration and blood collection, and rabbits are usually used to establish lesions of hyperlipidemia and atherosclerosis, which are rare in ischemic stroke models (Kumazawa et al., 2008).

Monkeys have a well-developed central nervous system, and their reactivity to drugs, functions, metabolism, blood biochemical characteristics, and cranial and brain development are extremely similar to those of humans, making them the most reliable animals for developing cerebral infarction models. In addition, they have a shorter reproduction cycle than humans. Sexually mature males are 3 years old, females are 2 years old, the pregnancy cycle is 5 months, and the life span is 20–30 years (Kobayashi et al., 2018). *Macaca fascicularis* in macaques reproduce relatively quickly compared to the others and are very light, weighing only 4–5 kg, and therefore it has a correspondingly small dose of drugs. Since the 1970s, most of the experimental monkeys have been used on *Macaca fascicularis*, especially in China, where they are also bred, accounting for almost 80–85% of the entire sector. Therefore, it is recommended to use *Macaca fascicularis* as experimental animals in future studies, which have their advantages in terms of price, number, and speed of reproduction. Some studies have shown that for *Macaca fascicularis*, males have greater body weight and absolute organ weights; however, for most organs, females account for a greater proportion of body weight (Amato et al., 2022). Thus, monkeys are suitable to develop the HIE models to further explore the effectiveness of drugs on HIE, but the monkeys-based investigations are limited by the fairly high price, scarce purchase sources and difficult breeding conditions. Although existing studies have modeled cerebral infarction in monkeys, the number of relevant monkey models studied in the last 30 years remains few (Crowell et al., 1981; Jones et al., 1981).

## 4. Overview of HIE

### 4.1. Pathophysiology of HI injury

**Figure 4:**
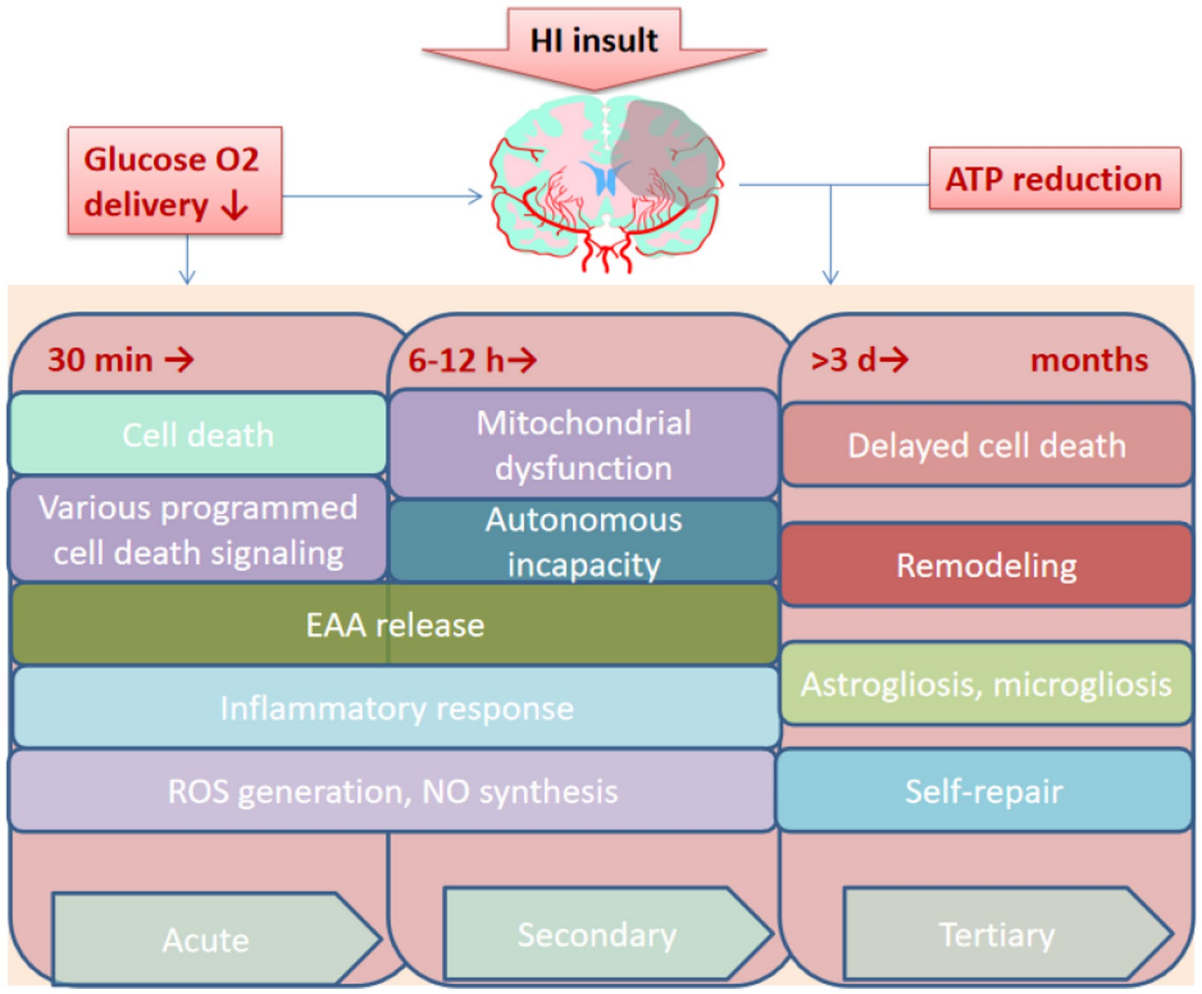
Overview of the pathological process of HIE. HI, Hypoxia-ischemia. EAA, Excitatory amino acid; ROS, Reactive oxygen species. NO, Nitric oxide; ATP, Adenosine triphosphate; min, minutes; h, hours; d, days.

HIE is caused by systemic asphyxia occurring at birth, and 25% of neonates with HIE develop severe and permanent neuropsychological sequelae, including Intellectual disability, cerebral palsy, and epilepsy (Taniguchi and Andreasson, 2008). Understanding the pathophysiology of injury during HI facilitates clinicians in the appropriate management of this critical condition in neonates, as the injury evolves over days or even weeks (Figure 4). The occurrence of HI disrupts the delivery of glucose in the umbilical cord due to a decrease in cardiac output and a reduction in cerebral blood flow. Decreased cerebral perfusion induces a cascade of events in distinct phases, which induces excitotoxicity and more cellular damage (Smyser et al., 2019). The acute or primary phase of injury lasts from 30 to 60 min after injury, whereas the latent phase may last from 1 to 6 h and is characterized by partial recovery and a continuingly activated apoptotic cascade (Wassink et al., 2014). Following the latent phase, the neonates suffer moderate to severe injury during the second phase of injury, which occurs within 6 to 15 h after injury and is manifested by cytotoxic edema, excitotoxicity, failure of mitochondrial activity, and cell death (Bennet et al., 2006). In the months after acute injury, the third phase involves delayed cell death, remodeling of the injured brain, and astrogliosis (Bennet et al., 2012). Despite emerging advances in supportive care, no effective treatments for HIE are currently available.

### 4.2. Distribution and dynamics of cerebral vessels in the brain

A fine and complete vascular system consisting of arteries, veins, and capillaries provides the structural basis for ensuring the blood supply (Hirsch et al., 2012). Alterations in the vascular structure can be found in many brain pathologies, and rupture of cerebral blood vessels causes severe vasospasm, which in turn may trigger ischemic stroke, usually in the vascular regions of the MCA and the anterior cerebral artery (ACA) (Gubskiy et al., 2018). Actually, in humans, the MCA and its branches are the most frequent cerebral vessels affected by stroke (up to 70% of all cerebral infarcts) (Bogousslavsky, 1988). Major rat cerebral arteries closely resemble their human counterparts in terms of the structure of the vascular wall and morphological changes associated with cerebral vascular diseases (Lee, 1995).

**Figure 5:**
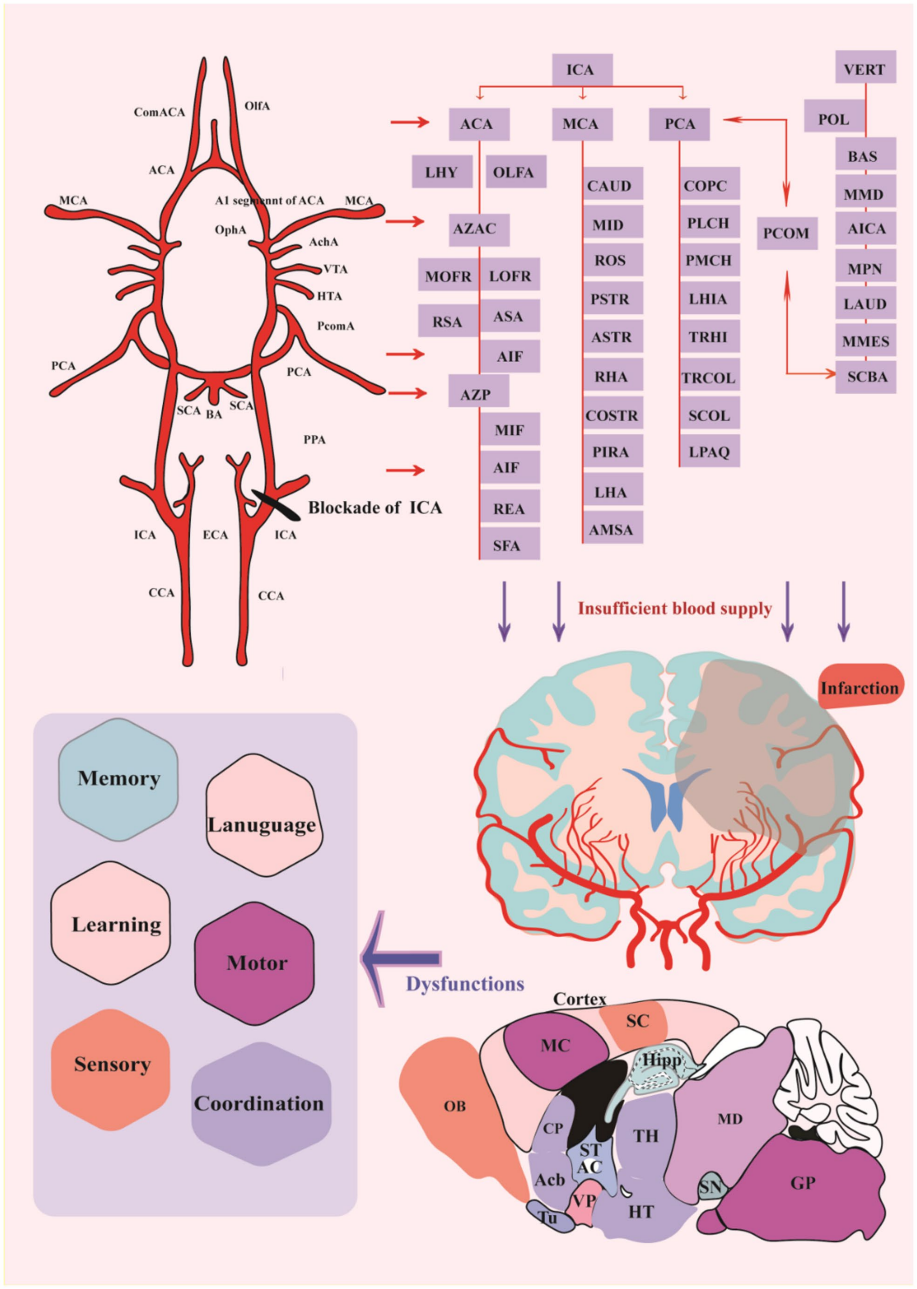
Distribution of cerebral vessels and blockade of crucial vessels cerebral infarction. ACA, anterior cerebral artery; AchA, anterior choroidal artery; BA, basilar artery; CCA, common carotid artery; ComACA, common (azygos) anterior cerebral artery; ECA, external carotid artery; HTA, hypothalamic artery; ICA, internal carotid artery; MCA, middle cerebral artery; OlfA, olfactory artery; OphA, ophthalmic artery; PCA, posterior cerebral artery; PcomA, posterior communicative artery; SCA, superior cerebellar artery; VTA, ventral thalamic artery; LHY, lateral hypothalamic artery; AZAC, Azygos of anterior cerebral artery; MOFR, Medial orbitofrontal artery; LOFR, Lateral orbitofrontal artery; RSA, Rostral septal artery; ASA, Ascending septal artery; AIF, Anterior internal frontal artery; AZP, Azygos pericallosal artery; MIF, Middle internal frontal artery; REA, Retrosplenial artery; SFA, Subfornical artery; CAUD, Caudal branch; MID, Middle branch; ROS, Rostral branch; PSTR, Posterior striate artery; ASTR, Anterior striate artery; RHA, Rhinal artery; COSTR, Corticostriate artery; PIRA, Piriform artery; LHA, Lateral hypothalamic artery; AMSA, Anterior medial striate artery; COPC, Cortical branches; PLCH, Posterior lateral choroidal artery; PMCH, Posterior medial choroidal artery; LHIA, Longitudinal hippocampal artery; TRHI, Transverse hippocampal artery; TRCOL, Transverse collicular artery; SCOL, Supracollicular arterial network; LPAQ, Lateral periaqueductal; VERT, Vertebral Artery; POL, Paraolivary artery; BAS, Basilar artery; MMD, Median medullary artery; AICA, Anterior inferior cerebellarl artery; MPN, Median pontine artery; LAUD, Internal auditory artery; MMES, Medial mesencephalic artery; SCBA, Superior Cerebellar artery. CP, caudate-putamen, dorsal striatum; Acb, nucleus accumbens; OB, Olfactory bulb; MC, motor cortex; SC, Somatosentory cortex; GP, Globus pallidus; Hipp, Hippocampus; ST, stria terminalis; AC, anterior commissure TH, Thalamus, HT, Hypothalamus; VP, ventral pallidum; Tu, Olfactory tubercle; SN, Substantial Nigra; MD, Mediodorsal thalamic nucleus.

The traced cerebrovascular system consists of six major arteries, including ACA, MCA, posterior cerebral artery (PCA), anterior choroidal artery (Ach) originating from the internal carotid Artery (ICA) and the basilar artery (BA), and superior cerebellar artery (SCA) (Courties et al., 2015), originated from the vertebral artery (VA) (Figure 5). The main trunks of ACA run along the midline region dorsal to the cortical surface, and its branches are mainly located in the hypothalamus (HT), STR, frontal lobes (Galasso et al., 2000), olfactory bulb (OLF), and dorsal part of the cerebral cortex. MCA is the middle branch of the ICA, and its branches within the brain are mainly located in the HT, CP, and amygdala (AMY) (Cotten et al., 2014). Ach is located in the caudal direction near the MCA and the branches of Ach in the ventral to dorsal direction, reaching the dorsum of the TH through the AMY, HT, TH, and choroid. PCA is the caudal branch of the ICA, and its trunk passes dorsally through the area between the cerebral cortex and midbrain (MB), with four large branches extending to the medial choroid, lateral choroid, ventral HIP, and somatosentory cortex (SC), respectively, while pial surface branches in the caudal to rostral direction supply the cerebral cortex not covered by the ACA and MCA. BA is located in the ventrum of the medulla and pons. Then, BA extends into the ventral part of the TH to form Scba, which extends in the ventral to dorsal direction into the rostral area of the cerebellum (CB). SCA supplies blood to the CB and is connected to the PCA via the posterior communicating artery (Pcom), forming cooperation between the two blood supply sources. In the rat MCAO models, the injury completely blocks direct blood flow from the ICA to the MCA and the reverse blood flow from the ACA (Gubskiy et al., 2018). And the A1 segment of ACA is the predominant injury site because it is thinner than the distal part of ICA, which bends to merge with the opposite ACA to form a common (azygos) ACA.

Furthermore, inadequate blood flow into the corresponding brain regions results in insufficient delivery of oxygen and glucose, further inducing tissue damage and loss. All post-stroke mice exhibit similar infarcts in the ipsilesional somatosensory cortex and STR, and some mice show extended infarcts in the ipsilesional TH and Hipp (Vahdat et al., 2021). Infarct in the discrete brain regions bring about specific dysfunctions in memory, learning, language, sensory, motor or coordination, etc. (Figure 5).

#### 4.2.1. Specific tissues loss and damaged regions after HIE

**Figure 6:**
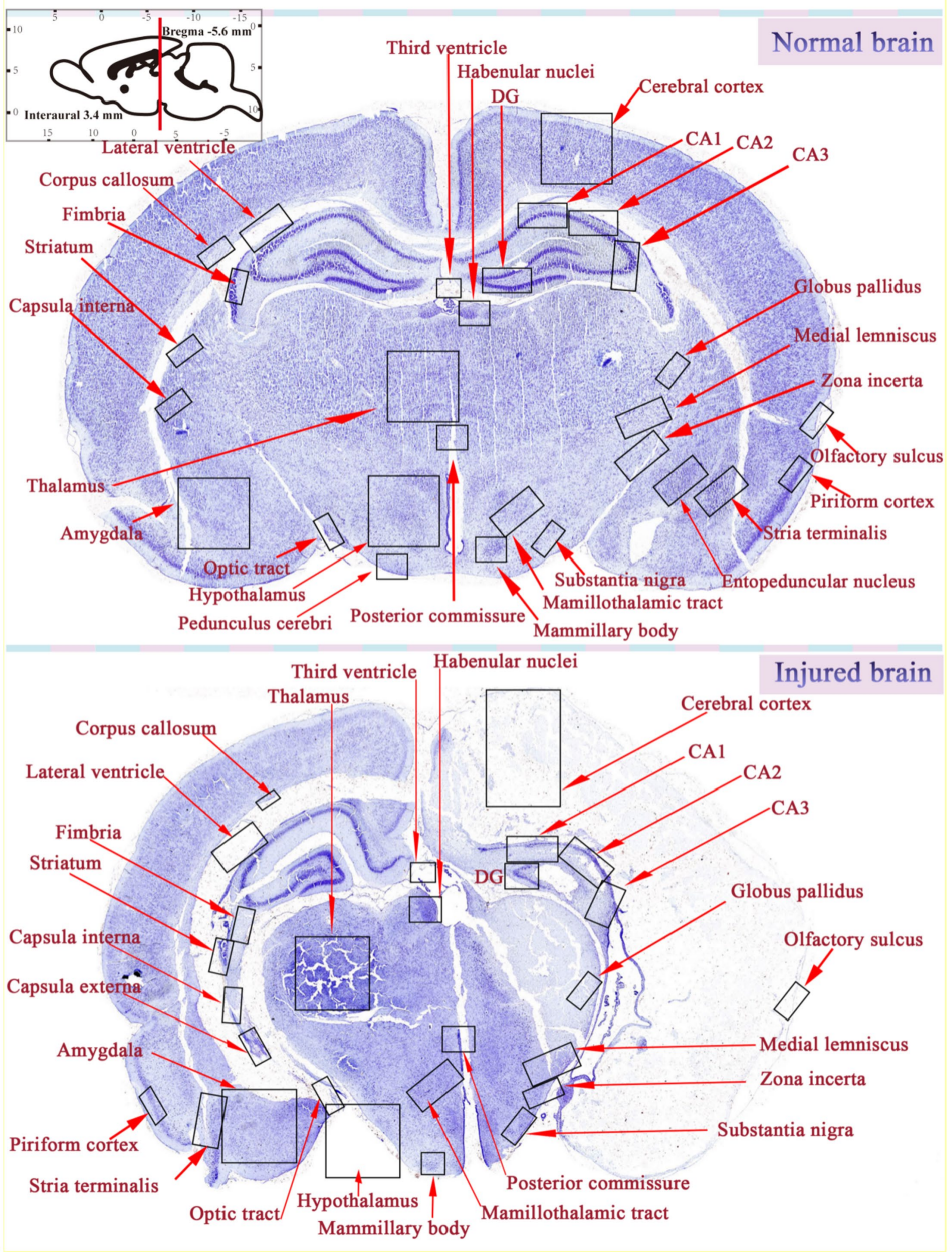
Comparison of brain areas between normal and damaged brains.

Blockade of cerebral blood vessels causes insufficient oxygen and glucose delivery, which in turn may induce infarction in the specified regions. Many studies have shown that HIE mainly affects the cerebral cortex, subcortical and periventricular white matter, STR (basal ganglia), and Hipp (Vannucci and Hagberg, 2004; Northington et al., 2005). Similarly, after comparing HIE rats with normal rats, we also found that brain injury in rats is mainly characterized by asymmetric atrophy of the injured hemisphere, and the key areas of the injury are mainly the cerebral cortex, TH, Hipp, and STR (Figure 6), manifesting as neurodegeneration, brain tissue infarction, and white matter loss. Moreover, morphological observations showed that some hyperchromatic cells and cytoplasmic vacuoles are formed in the Hipp and cortex of the ischemic side within a short time after injury, and a large number of cytoplasmic vacuolation and nuclear pyknosis in the ipsilateral neurons. Brain tissue loss was substantial, with most areas of the STR, Hipp, cortex, lateral thalamic nucleus, and posterior nucleus liquefied after 42 days (Tuor et al., 1998; Widerøe et al., 2011).

## 5. The role of neural circuits

**Figure 7:**
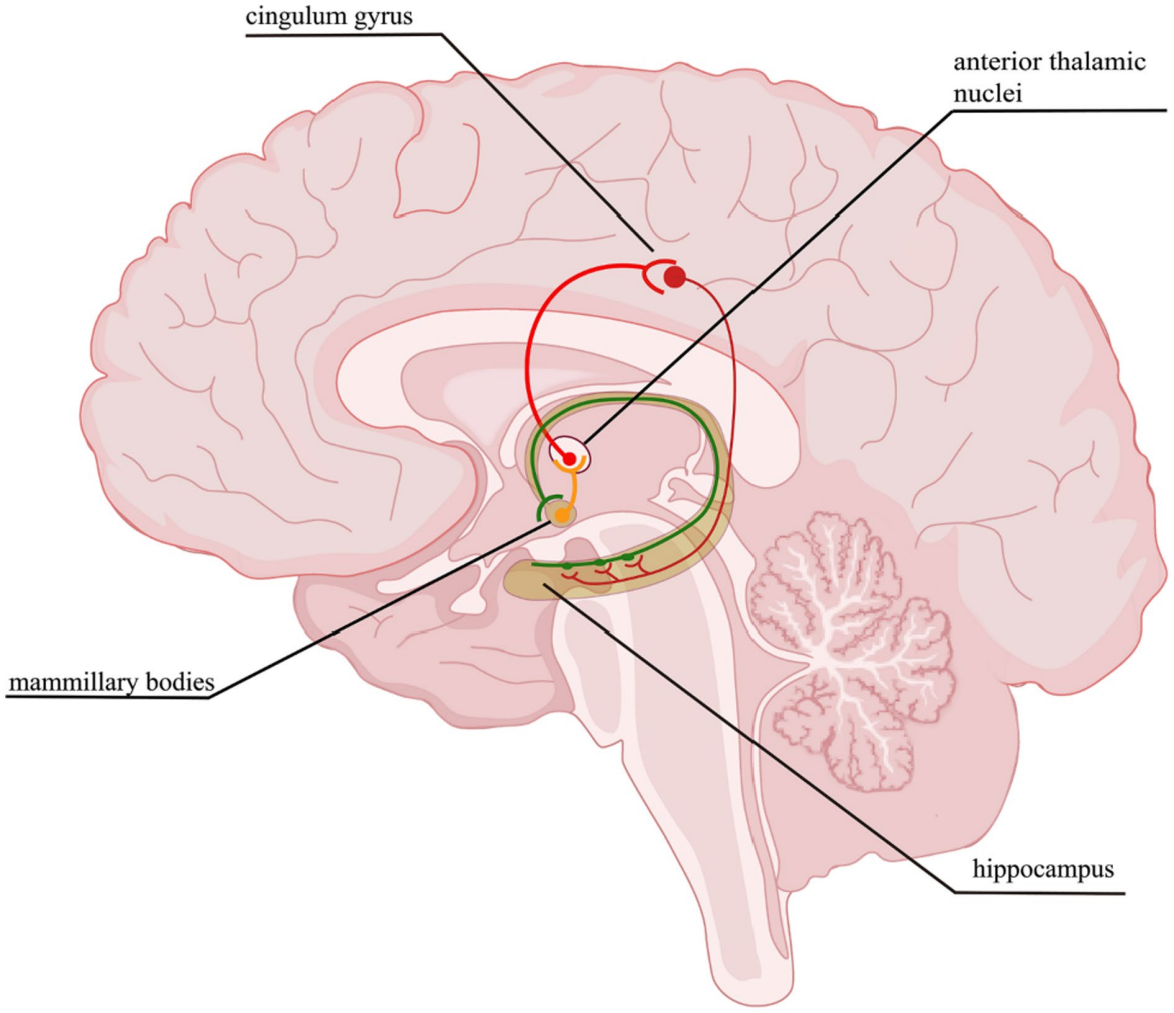
Papez circuit. The thalamocingulate tract connects the anterior thalamic nuclei through the cingulate cortex in the cingulum, then travels in a circuit through the parahippocampal gyrus and Hipp, mammillary bodies back to the anterior cingulate gyrus.

The cerebral cortex is an extremely complex system, containing approximately 86 billion neurons (Azevedo et al., 2009). The interconnected neurons form neural circuits between nerve cells with defined functions, forming complex neural networks that are the basis of the brain’s nervous system. A neural circuit is a trisynaptic structure consisting of axons, dendritic ends, and glial cells. Neurons establish connections through synapses, thus forming extremely complex neural circuits for information transmission and processing to realize brain function. Following its role in synaptic connection, cortical neurons can be mainly divided into excitatory and inhibitory neurons (interneurons). Although interneurons account for only about 20% of the total cortical neurons, they play a vital role in the function of neuronal circuits (Ozawa and Johansen, 2014). Many scholars have found that the occurrence and development of a variety of neurological diseases, such as epilepsy (Duchowny et al., 2000), autism (Chen et al., 2018; Fernández et al., 2018; Di et al., 2020; Liang et al., 2021), schizophrenia (Laviolette, 2007; Wang et al., 2021), and bipolar disorder (Shi et al., 2020; Rai et al., 2021), are closely related to the establishment and regulation of neural circuits. Therefore, investigations targeting neural circuits exert an important role in the pathogenesis, diagnosis, and treatment of many diseases. The Papez circuit (Figure 7), which mainly consists of the mammillary bodies, anterior thalamic nucleus and Hipp, is the key to memory (Nishio et al., 2014). The brain areas damaged after HIE include the Hipp and TH, and not only that, there are a large number of neural circuits in both the cerebral cortex and STR and when these are disrupted, the corresponding functions are also destroyed, for instance, the motion, memory (Yamamoto et al., 2021), cognitive function (Yan et al., 2022), muscle tone, etc. It has been shown that abnormal thalamocortical input may affect the organization and function of cortical neuronal circuits, especially if they are in a developmental window period (Shoykhet and Middleton, 2016), however, HIE often occurs in neonates when they are in a critical period of growth and development. Researchers have mapped the neural circuits in response to deep brain stimulation in the anterior thalamic nucleus of the rat brain used to treat epilepsy (Yang et al., 2022), but there is a gap in the current study of neural circuits in HIE that warrants further study.

### 5.1. Neurons projections across brain regions

**Figure 8:**
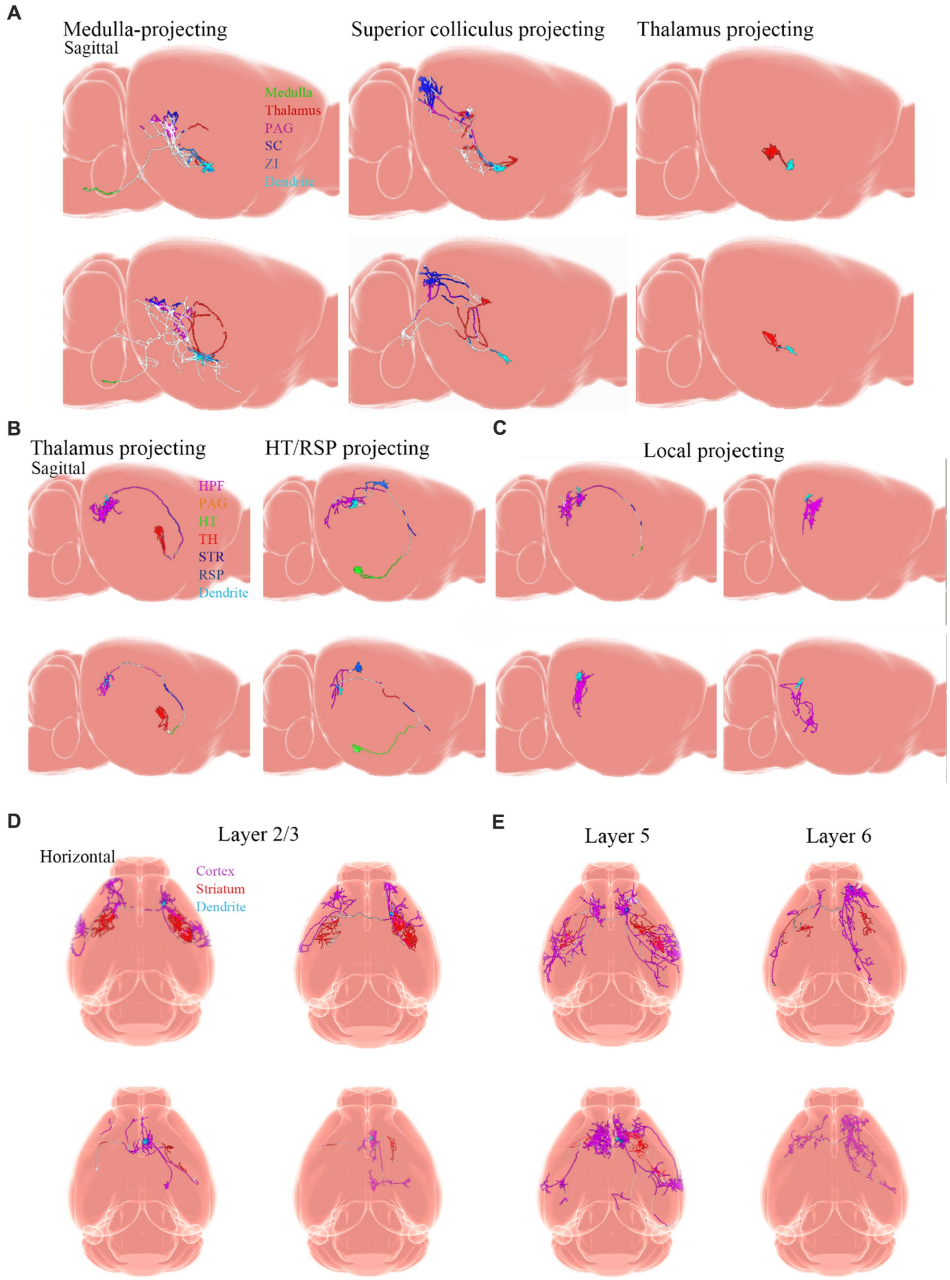
Projections from neurons. Adapted from Winnubst et al. (2019). **(A)** Examples of single ZI neurons. Axons are color-coded according to anatomical position. Dendrites are shown in light blue. **(B)** Thalamus-projecting neurons, HT/RSP projecting neurons, and **(C)** local-projecting neurons. Inset shows the long-range axonal end of a local-projecting neuron that lacks varicosities. **(D,E)** Horizontal view of individual MC IT neurons with axons color-coded according to their anatomical position. Dendrites are shown in light blue. PAG, periaqueductal gray; SCC, superior colliculus; ZI, zona incerta; HPF, hippocampal formation; HT, hypothalamus; TH, thalamus; STR, striatum; RSP, retrosplenial cortex.

**Figure 9:**
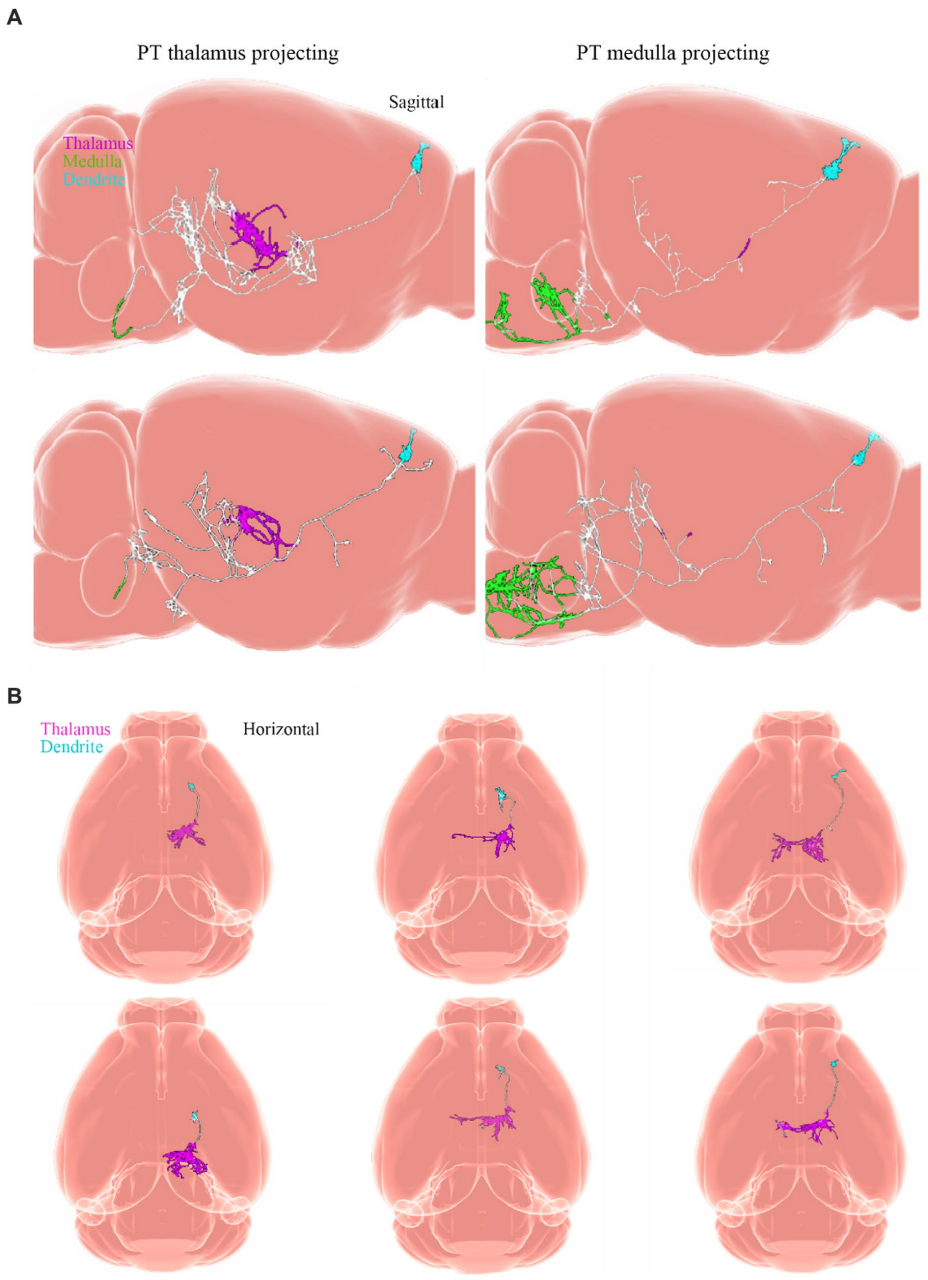
Projections from neurons. Adapted from Winnubst et al. (2019). **(A)** Sagittal view of single PT neurons that project to the thalamus (PT thalamus-projecting; magenta) or medulla (PT medulla-projecting; green) respectively. **(B)** Horizontal view of single layer 6 corticothalamic neurons and their axonal projections to the TH (magenta). Dendrites are light blue. PT, pyramidal tract.

Neuronal cell types are the nodes of neural circuits that determine the information flow within the brain. Mammalian neurons possess extensive axonal projections that extend over long distances across all major brain structures. These projections dictate how information flows across brain areas, and it is crucial to get knowledge of neurons primarily from the zona incerta (ZI), TH, Hipp, cerebral cortex, and HT, as well as their distributed projections. ZI is associated with a variety of functions, including defensive, behavior, appetite, and sensory gating (Chou et al., 2018). In the dataset of Allen Mouse Common Coordinate Framework, ZI neurons project to the periaqueductal gray, superior colliculus, ZI, and TH (Figure 8A), and subiculum neurons project to the hippocampal formation (HPF), periaqueductal gray, HT, TH, STR, and retrosplenial cortex (RSP) (Figure 8B). Innervation of the STR is confined mostly to the lateral septum and Acb. Besides, projection neurons in the cerebral cortex are deemed to have complex axonal arborizations (Figures 8B,C). Allen Mouse Common Coordinate Framework contains MC neurons, including intratelencephalic (IT) neurons in layers 2–6, pyramidal tract (PT) (Bhatia et al., 2018) neurons in layer 5b, and corticothalamic (CT) neurons in layer 6 (Winnubst et al., 2019). IT neurons in the database reveal projections that were limited mostly to the cortex and STR, with fewer projections to the basolateral amygdala and the claustrum, and the vast majority of IT neurons project to the STR and across the corpus callosum (Figures 8D,E). The proportion of axonal length in the STR or cortex varies by more than one order of magnitude and does not depend on the size of the axonal arbor. Individual IT neurons can have up to 40 cm of axon and project to many distinct cortical areas and the STR. In addition, neurons in the ventral anterior-lateral complex of the TH project to a large area of the ipsilateral MC (Bosch-Bouju et al., 2013), and additionally project to the somatosensory cortex. Some neurons are also a branch in the reticular nucleus and/or the dorsal striatum (Figures 9A,B).

## 6. Advances in neural circuits related to technology and applications

In the past, research on HIE has focused on behavioral, morphological, and molecular mechanisms and very little research has been performed on neural circuit mechanisms. However, with the development of scientific techniques electrical stimulation ques such as calcium imaging, optogenetics and chemogenetic, it is believed that the study of neural circuits is becoming a hot topic. Subsequently, we described the progress and applications of neural circuit-related technologies in recent years.

### 6.1. Techniques used for electrical stimulation on neural circuits

Brain stimulation techniques, such as electrical stimulation and transcranial magnetic stimulation, have been used to directly change circuit activity to promote functional recovery and to study circuit remapping (Ward and Cohen, 2004; Pekna et al., 2012). However, these techniques non-selectively target all cell types near the stimulation site, such as neurons, glial cells, and oligodendrocytes, so it is difficult to study potential cell types and circuit mechanisms driving post-stroke recovery (Gradinaru et al., 2009; Takatsuru et al., 2013). For these reasons, papers on electrical stimulation to study neural circuits have declined in recent years, while emerging techniques such as calcium imaging are becoming the dominant method for studying neural circuits due to their advantages.

### 6.2. Techniques used for calcium imaging on neural circuits

In the process of analyzing the regulation mechanism of neural circuits, calcium imaging technology can accurately reflect the neuronal activity in the brain during the motor process by monitoring the changes of Ca^2+^ in real-time. It has become a routine technology to monitor and analyze the functional activity of neurons in recent years, and it is constantly being updated and improved. In a recent study, researchers applied *in vivo* calcium imaging to the macaque brain and developed miniature fluorescence microscopy to image the primary visual cortex of the macaque, and dozens of clear fluorescent signals were successfully identified in the macaque brain hemisphere. Eventually, a subset of these neurons showed clear retinal localization and orientation adjustments and stimulus orientation was successfully decoded and the cells were tracked, demonstrating that microendoscopic calcium imaging is feasible and reasonable for studying neural circuits in the macaque brain by monitoring fluorescent signals from a large number of neurons (Oguchi et al., 2021). Moreover, it has been noted that the combination of molecular genetic techniques and calcium imaging allows for real-time observation of complex neural circuits, which is superior to electrophysiological methods, and it is less invasive as it does not rupture or puncture the cell membrane as electrodes do (Luo, 2021).

### 6.3. Techniques used for optogenetics on neural circuits

Optical imaging plays an essential role in describing the reorganization pattern of cortical circuits *in vivo* after stroke. Sensory remodeling after stroke is accompanied by abnormally prolonged sensory-evoked responses and changes in connection patterns of periinfarct areas and further areas (Brown et al., 2009). The Optogenetics technique is a fusion of the two fields of optics and genetics, and optogenetics is used to manipulate specific neurons/circuits *in vivo* to detect the temporal and spatial changes in the whole-brain neural circuits (cortical and subcortical circuits) recovered after stroke (Lee et al., 2010; Vahdat et al., 2021). Using this unique method, optogenetic neuronal stimulation in the ipsilateral primary motor cortex (ipsilateral lesion) (Cheng et al., 2014), thalamic cortical circuits (Tennant et al., 2017), contralateral corticospinal tract (Wahl et al., 2017), and contralateral deep cerebellar nucleus can promote functional recovery after stroke (Shah et al., 2017; Vahdat et al., 2021). This method provides functional activation through cell type-specific regulation of neural circuits but also can be used to analyze the basic mechanism of designated stem cells in the functional recovery of HIE. At the same time, a technique called optogenetic functional magnetic resonance imaging (ofMRI) has appeared (Lin et al., 2016). This novel approach combines optogenetic stimulation and functional magnetic resonance imaging, where the dynamics of functional brain circuits of the same animal can be tracked unbiasedly and longitudinally during recovery.

### 6.4. Techniques used for chemogenetic on neural circuits

The chemogenetic technique consists of two parts, a combination of chemical-based drugs and gene technology that uses genetic technology to modify proteins in organisms to control the activity of small biomolecules. Using chemical genetic techniques to bidirectionally modulate neuronal activity between the ACC and the ventral hippocampus, the neural circuit from the ACC to the ventral hippocampus is eventually found to control the expression of fear generalization. This finding suggests new possibilities for the prevention and treatment of contextual fear generalization in patients with anxiety disorders, particularly in patients with post-traumatic stress disorder (Bian et al., 2019).

## 7. Conclusion and prospects

### 7.1. Future emphasis on large animal studies for HIE treatment

Exploration of HIE treatment presents a daunting task in both the preclinical and clinical fields. In the course of investigations, various factors do matter, including the types of animals selected for mimicking human conditions, the appropriate age, and the proper time points for such experiments as behavioral and histological evaluations. Generally, mice and rats have been used widely to establish HIE models, besides, non-rodents are quite representative of neonatal HI to further simulate the clinical symptoms of human patients, but a minority of research has investigated larger animals such as pigs, sheep, or macaques. For example, macaque fetuses, pigs, and fetal sheep all have been employed to establish the HI models. Other small experimental animals, such as preterm rabbit fetuses, have also been subjected to intrauterine occlusion of the descending aorta to induce HI, and significant alterations can be shown in motor responses to olfactory stimuli, coordination of sucking and swallowing, etc. Comparatively, large animals own the advantages of replicating the conditions of a single human fetus exposed to a non-sterile environment, allowing a better comparison of brain development at birth. Among these animals, macaques belong to non-human primates with advanced brain function, brain anatomical structure, physiological metabolism, and immune system, and many biological characteristics such as the sleep rhythm are similar to those of humans, and with a shorter reproduction cycle than humans. Overall, this will help us to understand the efficiency of potential therapeutics to save animals from HIE for future applications in humans. It has been reported that animals of different sex respond differently to HIE and different treatments, with females exhibiting better neurological performance due to the neuroprotective role of estrogens, such as cell genesis or an innate immune response secondary to the primary injury contributing to sexual dimorphism in HIE (El-Khodor and Boksa, 2003; Mirza et al., 2015; Chanana et al., 2016; Waddell et al., 2016). Therefore, it is also important to observe and compare the neurological reactions between male and female animals. The choice of animal models in the future could focus on non-human primates, such as macaques, where it has been demonstrated to be feasible and reasonable to study the neural circuits of the monkey brain by calcium imaging (Oguchi et al., 2021).

### 7.2. Damage to brain areas following HIE

HIE will remain a major cause of severe and permanent neuropsychological sequelae of neonates. The researchers found significantly more cellular damage in the ipsilateral cortical, CA1 pyramidal layer, and hippocampal dentate gyrus (DG) in ipsilateral hypoxic/ischemic tissue compared to normal tissue (Olson and McKeon, 2004). In addition, many studies have shown that HIE mainly affects the cerebral cortex, subcortical and periventricular white matter, STR (basal ganglia), and Hipp (Vannucci and Hagberg, 2004; Northington et al., 2005). To our knowledge, ischemic damage distinctively affects the sub-regions of the Hipp; for example, the hippocampal CA1 region is more sensitized to ischemic injury and neurodegeneration (Chen et al., 1996), whereas neurogesis is usually activated within the DG area (Jin et al., 2001). Moreover, it has been reported that cerebral WMI disrupts the normal progression of developmental myelination and causes many chronic neurological morbidities from cerebral palsy and diverse neurobehavioral disabilities (Back, 2017). Accordingly, in the future, researchers need to focus on the tissue-specific mechanisms in the investigations of neural circuit therapy for HIE treatment.

### 7.3. Future emphasis on neurocircuitry research for HIE treatment

With the advent of calcium imaging and optogenetics and chemogenetic, which are constantly updated and developed, providing unprecedented opportunities for the study of neural circuits. Perhaps the study of neural circuits can reveal new insights into the treatment of HIE. Firstly, the application of neuromodulation strategies for HIE is important as key neural circuits spanning different brain regions are associated with functional recovery, and further advances in the treatment of HIE may be achieved through specific modulation of neural circuits. Secondly, damage to the nervous system can lead not only to damage to brain regions but also to the absence of neural circuits, resulting in the loss or disruption of functions regulated by neural circuits. So in the future, the formation and remodeling of neural circuits can be further investigated to restore function.

### 7.4. Other new research methods

At present, the research on nervous system diseases mainly relies on the construction of disease animal models or clinical studies. However, both methods have their limitations. It is difficult for clinical research to start from the early stage of disease development, and the use of animal models has the problems of species differences and cannot reproduce the specific process of humans. In recent years, *in vitro*, models derived from 3D cultured human pluripotent stem cells are rapidly developing, and some breakthroughs have been made in organoids such as the brain, heart, and lung. Many studies have used human pluripotent stem cell-derived brain organoids to elucidate the mysteries of human brain development and to model neurological diseases such as Alzheimer’s disease (Barak et al., 2022; Bubnys and Tsai, 2022), autism spectrum disorders (Chan et al., 2020). Malformations of human cortical development (Jabali et al., 2022), microcephaly (Jayaraman et al., 2018). Various central nervous system virus infections (Depla et al., 2022). There have been scholars using organoids for Neonatal hypoxic injury studies not long ago (Boisvert et al., 2019). This novel approach in the neuroscience Toolbox provides new ideas for probing human neurodevelopmental and disease processes, with which to better understand human-specific processes in the nervous system development and disease, with landmark implications for the study of neurological disorders. Moreover, as a new technology, single-cell sequencing can reflect the nature of cellular unit life, deepen the understanding of various biological manifestations of the disease, help reveal the pathogenesis of HIE, and provide new ideas for clinical diagnosis and treatment (Chen et al., 2021).

## Author contributions

H-QS: original draft preparation, visualization, review and editing. Y-FS: review and editing. LC and H-SZ: writing—original draft preparation. Q-XX: writing—review and editing. B-YL: review and editing. DZ and Q-YC: visualization. L-LX: conceptualization. All authors contributed to the article and approved the submitted version.

## Funding

This work was supported by the National Natural Science Foundation of China (no. 82001604); Guizhou Provincial Basic Research Program (Natural Science) (ZK [2021]-368); Collaborative Innovation Center of Chinese Ministry of Education (2020-39); and Zunyi Medical University 12345 Future Talent Training Program - Technology Elite grant number (ZYSE-2021-03).

## Conflict of interest

The authors declare that the research was conducted in the absence of any commercial or financial relationships that could be construed as a potential conflict of interest.

The reviewer XT declared a shared affiliation with the authors LC and Q-XX to the handling editor at the time of review.

## Publisher’s note

All claims expressed in this article are solely those of the authors and do not necessarily represent those of their affiliated organizations, or those of the publisher, the editors and the reviewers. Any product that may be evaluated in this article, or claim that may be made by its manufacturer, is not guaranteed or endorsed by the publisher.

## References

[ref1] Alger J. R. Brunetti A. Nagashima G. Hossmann K. A. (1989). Assessment of postischemic cerebral energy metabolism in cat by 31P NMR: the cumulative effects of secondary hypoxia and ischemia. J. Cereb. Blood Flow Metab. 9, 506–514. doi: 10.1038/jcbfm.1989.74, PMID: 2738116

[ref2] Amato R. Gardin J. F. Tooze J. A. Cline J. M. (2022). Organ weights in relation to age and sex in Cynomolgus monkeys (*Macaca fascicularis*). Toxicol. Pathol. 50, 574–590. doi: 10.1177/01926233221088283, PMID: 35383510 PMC9308629

[ref3] Arteni N. S. Salgueiro J. Torres I. Achaval M. Netto C. A. (2003). Neonatal cerebral hypoxia-ischemia causes lateralized memory impairments in the adult rat. Brain Res. 973, 171–178. doi: 10.1016/s0006-8993(03)02436-3, PMID: 12738060

[ref4] Azevedo F. A. Carvalho L. R. Grinberg L. T. Farfel J. M. Ferretti R. E. Leite R. E.. (2009). Equal numbers of neuronal and nonneuronal cells make the human brain an isometrically scaled-up primate brain. J. Comp. Neurol. 513, 532–541. doi: 10.1002/cne.21974, PMID: 19226510

[ref5] Back S. A. (2017). White matter injury in the preterm infant: pathology and mechanisms. Acta Neuropathol. 134, 331–349. doi: 10.1007/s00401-017-1718-6, PMID: 28534077 PMC5973818

[ref6] Barak M. Fedorova V. Pospisilova V. Raska J. Vochyanova S. Sedmik J.. (2022). Human iPSC-derived neural models for studying Alzheimer’s disease: from neural stem cells to cerebral organoids. Stem Cell Rev. Rep. 18, 792–820. doi: 10.1007/s12015-021-10254-3, PMID: 35107767 PMC8930932

[ref7] Bennet L. Roelfsema V. Pathipati P. Quaedackers J. S. Gunn A. J. (2006). Relationship between evolving epileptiform activity and delayed loss of mitochondrial activity after asphyxia measured by near-infrared spectroscopy in preterm fetal sheep. J. Physiol. 572, 141–154. doi: 10.1113/jphysiol.2006.105197, PMID: 16484298 PMC1779651

[ref8] Bennet L. Tan S. Van den Heuij L. Derrick M. Groenendaal F. van Bel F.. (2012). Cell therapy for neonatal hypoxia-ischemia and cerebral palsy. Ann. Neurol. 71, 589–600. doi: 10.1002/ana.22670 22522476

[ref9] Bhatia V. Gupta V. Khurana D. Sharma R. R. Khandelwal N. (2018). Randomized assessment of the safety and efficacy of intra-arterial infusion of autologous stem cells in subacute ischemic stroke. AJNR Am. J. Neuroradiol. 39, 899–904. doi: 10.3174/ajnr.A5586, PMID: 29545253 PMC7410650

[ref10] Bian X. L. Qin C. Cai C. Y. Zhou Y. Tao Y. Lin Y. H.. (2019). Anterior cingulate cortex to ventral Hippocampus circuit mediates contextual fear generalization. J. Neurosci. 39, 5728–5739. doi: 10.1523/JNEUROSCI.2739-18.2019, PMID: 31097621 PMC6636085

[ref11] Bogousslavsky J. (1988). Leukoencephalopathy, leukoaraiosis and cerebral infarction. Rev. Neurol. (Paris) 1441, 11–17. 3279483

[ref12] Boisvert E. M. Means R. E. Michaud M. Madri J. A. Katz S. G. (2019). Minocycline mitigates the effect of neonatal hypoxic insult on human brain organoids. Cell Death Dis. 10:325. doi: 10.1038/s41419-019-1553-x, PMID: 30975982 PMC6459920

[ref13] Bosch-Bouju C. Hyland B. I. Parr-Brownlie L. C. (2013). Motor thalamus integration of cortical, cerebellar and basal ganglia information: implications for normal and parkinsonian conditions. Front. Comput. Neurosci. 7:163. doi: 10.3389/fncom.2013.00163, PMID: 24273509 PMC3822295

[ref14] Brown C. E. Aminoltejari K. Erb H. Winship I. R. Murphy T. H. (2009). In vivo voltage-sensitive dye imaging in adult mice reveals that somatosensory maps lost to stroke are replaced over weeks by new structural and functional circuits with prolonged modes of activation within both the peri-infarct zone and distant sites. J. Neurosci. 29, 1719–1734. doi: 10.1523/jneurosci.4249-08.2009, PMID: 19211879 PMC6666293

[ref15] Bubnys A. Tsai L. H. (2022). Harnessing cerebral organoids for Alzheimer's disease research. Curr. Opin. Neurobiol. 72, 120–130. doi: 10.1016/j.conb.2021.10.003, PMID: 34818608

[ref16] Chan W. K. Griffiths R. Price D. J. Mason J. O. (2020). Cerebral organoids as tools to identify the developmental roots of autism. Mol. Autism. 11:58. doi: 10.1186/s13229-020-00360-3, PMID: 32660622 PMC7359249

[ref17] Chan D. Suk H. J. Jackson B. Milman N. P. Stark D. Beach S. D.. (2021). Induction of specific brain oscillations may restore neural circuits and be used for the treatment of Alzheimer's disease. J. Intern. Med. 290, 993–1009. doi: 10.1111/joim.13329, PMID: 34156133

[ref18] Chanana V. Tumturk A. Kintner D. Udho E. Ferrazzano P. Cengiz P. (2016). Sex differences in mouse hippocampal astrocytes after in-vitro ischemia. J. Vis. Exp. 116:53695. doi: 10.3791/53695, PMID: 27805577 PMC5092229

[ref19] Chavan S. S. Tracey K. J. (2014). Regulating innate immunity with dopamine and electroacupuncture. Nat. Med. 20, 239–241. doi: 10.1038/nm.3501, PMID: 24603793

[ref20] Chen H. Wang J. Uddin L. Q. Wang X. Guo X. Lu F.. (2018). Aberrant functional connectivity of neural circuits associated with social and sensorimotor deficits in young children with autism spectrum disorder. Autism Res. 11, 1643–1652. doi: 10.1002/aur.2029, PMID: 30475453 PMC6281874

[ref21] Chen J. Zhu R. L. Nakayama M. Kawaguchi K. Jin K. Stetler R. A.. (1996). Expression of the apoptosis-effector gene, Bax, is up-regulated in vulnerable hippocampal CA1 neurons following global ischemia. J. Neurochem. 67, 64–71. doi: 10.1046/j.1471-4159.1996.67010064.x, PMID: 8667027

[ref190] Chen T. B. Zhou H. S. Xiong L. L. (2021). Single cell sequencing technology and its application in Hypoxic ischemic encephalopathy research. Ibrain. 73, 227–234. doi: 10.1002/j.2769-2795.2021.tb00086.x, PMID: 37786794 PMC10528982

[ref22] Cheng M. Y. Wang E. H. Woodson W. J. Wang S. Sun G. Lee A. G.. (2014). Optogenetic neuronal stimulation promotes functional recovery after stroke. Proc. Natl. Acad. Sci. U. S. A. 111, 12913–12918. doi: 10.1073/pnas.1404109111, PMID: 25136109 PMC4156770

[ref23] Chou X. L. Wang X. Zhang Z. G. Shen L. Zingg B. Huang J.. (2018). Inhibitory gain modulation of defense behaviors by zona incerta. Nat. Commun. 9:1151. doi: 10.1038/s41467-018-03581-6, PMID: 29559622 PMC5861117

[ref24] Cotten C. M. Murtha A. P. Goldberg R. N. Grotegut C. A. Smith P. B. Goldstein R. F.. (2014). Feasibility of autologous cord blood cells for infants with hypoxic-ischemic encephalopathy. J. Pediatr. 164, 973–979.e1. doi: 10.1016/j.jpeds.2013.11.036, PMID: 24388332 PMC3992180

[ref25] Courties G. Herisson F. Sager H. B. Heidt T. Ye Y. Wei Y.. (2015). Ischemic stroke activates hematopoietic bone marrow stem cells. Circ. Res. 116, 407–417. doi: 10.1161/circresaha.116.305207, PMID: 25362208 PMC4312511

[ref26] Crowell R. M. Marcoux F. W. DeGirolami U. (1981). Variability and reversibility of focal cerebral ischemia in unanesthetized monkeys. Neurology 31, 1295–1302. doi: 10.1212/wnl.31.10.1295, PMID: 7202140

[ref27] Dean J. M. Shi Z. Fleiss B. Gunn K. C. Groenendaal F. van Bel F.. (2015). A critical review of models of perinatal infection. Dev. Neurosci. 37, 289–304. doi: 10.1159/000370309 25720344

[ref28] Depla J. A. Mulder L. A. de Sá R. V. Wartel M. Sridhar A. Evers M. M.. (2022). Human brain organoids as models for central nervous system viral infection. Viruses 14:634. doi: 10.3390/v14030634, PMID: 35337041 PMC8948955

[ref29] Di J. Li J. O'Hara B. Alberts I. Xiong L. Li J.. (2020). The role of GABAergic neural circuits in the pathogenesis of autism spectrum disorder. Int. J. Dev. Neurosci. 80, 73–85. doi: 10.1002/jdn.10005, PMID: 31910289

[ref30] Duchowny M. Jayakar P. Levin B. (2000). Aberrant neural circuits in malformations of cortical development and focal epilepsy. Neurology 55, 423–428. doi: 10.1212/wnl.55.3.423, PMID: 10932280

[ref31] Durukan A. Tatlisumak T. (2007). Acute ischemic stroke: overview of major experimental rodent models, pathophysiology, and therapy of focal cerebral ischemia. Pharmacol. Biochem. Behav. 87, 179–197. doi: 10.1016/j.pbb.2007.04.015, PMID: 17521716

[ref32] El-Khodor B. F. Boksa P. (2003). Differential vulnerability of male versus female rats to long-term effects of birth insult on brain catecholamine levels. Exp. Neurol. 182, 208–219. doi: 10.1016/s0014-4886(03)00079-7, PMID: 12821391

[ref33] Feng Y. Zhu G. Chen R. Shi G. Peng M. Zhou Y.. (2022). Electroacupuncture remodels the extracellular matrix and promotes synaptic plasticity in a mouse model of depression. Biochem. Biophys. Res. Commun. 626, 44–50. doi: 10.1016/j.bbrc.2022.07.077, PMID: 35970043

[ref34] Fernández M. Mollinedo-Gajate I. Peñagarikano O. (2018). Neural circuits for social cognition: implications for autism. Neuroscience 370, 148–162. doi: 10.1016/j.neuroscience.2017.07.013, PMID: 28729065

[ref35] Galasso J. M. Liu Y. Szaflarski J. Warren J. S. Silverstein F. S. (2000). Monocyte chemoattractant protein-1 is a mediator of acute excitotoxic injury in neonatal rat brain. Neuroscience 101, 737–744. doi: 10.1016/s0306-4522(00)00399-7, PMID: 11113322

[ref36] Garberg H. T. Solberg R. Barlinn J. Martinez-Orgado J. Løberg E. M. Saugstad O. D. (2017). High-dose cannabidiol induced hypotension after global hypoxia-ischemia in piglets. Neonatology 112, 143–149. doi: 10.1159/000471786, PMID: 28564654

[ref37] Gradinaru V. Mogri M. Thompson K. R. Henderson J. M. Deisseroth K. (2009). Optical deconstruction of parkinsonian neural circuitry. Science 324, 354–359. doi: 10.1126/science.1167093, PMID: 19299587 PMC6744370

[ref350] Guan Y. H. Zhou H. S. Luo B. Y. Hussain S. Xiong L. L. (2023). Research progress of neonatal hypoxic‐ischemic encephalopathy in nonhuman primate models. Ibrain. doi: 10.1002/ibra.12097, PMID: 37786551 PMC10528769

[ref38] Gubskiy I. L. Namestnikova D. D. Cherkashova E. A. Chekhonin V. P. Baklaushev V. P. Gubsky L. V.. (2018). MRI guiding of the middle cerebral artery occlusion in rats aimed to improve stroke modeling. Transl. Stroke Res. 9, 417–425. doi: 10.1007/s12975-017-0590-y, PMID: 29178027 PMC6061245

[ref39] Hermann D. M. Popa-Wagner A. Kleinschnitz C. Doeppner T. R. (2019). Animal models of ischemic stroke and their impact on drug discovery. Expert Opin Drug Discov. 14, 315–326. doi: 10.1080/17460441.2019.1573984, PMID: 30714423

[ref40] Hirsch S. Reichold J. Schneider M. Székely G. Weber B. (2012). Topology and hemodynamics of the cortical cerebrovascular system. J. Cereb. Blood Flow Metab. 32, 952–967. doi: 10.1038/jcbfm.2012.39, PMID: 22472613 PMC3367227

[ref41] Jabali A. Hoffrichter A. Uzquiano A. Marsoner F. Wilkens R. Siekmann M.. (2022). Human cerebral organoids reveal progenitor pathology in EML1-linked cortical malformation. EMBO Rep. 23:e54027. doi: 10.15252/embr.202154027, PMID: 35289477 PMC9066063

[ref42] Jayaraman D. Bae B. I. Walsh C. A. (2018). The genetics of primary microcephaly. Annu. Rev. Genomics Hum. Genet. 19, 177–200. doi: 10.1146/annurev-genom-083117-021441 29799801

[ref43] Jin K. Minami M. Lan J. Q. Mao X. O. Batteur S. Simon R. P.. (2001). Neurogenesis in dentate subgranular zone and rostral subventricular zone after focal cerebral ischemia in the rat. Proc. Natl. Acad. Sci. U. S. A. 98, 4710–4715. doi: 10.1073/pnas.081011098, PMID: 11296300 PMC31899

[ref44] Jones T. H. Morawetz R. B. Crowell R. M. Marcoux F. W. FitzGibbon S. J. DeGirolami U.. (1981). Thresholds of focal cerebral ischemia in awake monkeys. J. Neurosurg. 54, 773–782. doi: 10.3171/jns.1981.54.6.0773, PMID: 7241187

[ref45] Kletkiewicz H. Klimiuk M. Woźniak A. Mila-Kierzenkowska C. Dokladny K. Rogalska J. (2020). How to improve the antioxidant defense in asphyxiated newborns-lessons from animal models. Antioxidants 9:898. doi: 10.3390/antiox9090898, PMID: 32967335 PMC7554981

[ref46] Kobayashi M. Koyama T. Yasutomi Y. Sankai T. (2018). Relationship between menarche and fertility in long-tailed macaques (*Macaca fascicularis*). J. Reprod. Dev. 64, 337–342. doi: 10.1262/jrd.2017-164, PMID: 29848903 PMC6105741

[ref47] Kumazawa M. Iida H. Uchida M. Iida M. Takenaka M. Fukuoka N.. (2008). The effects of transient cerebral ischemia on vasopressin-induced vasoconstriction in rabbit cerebral vessels. Anesth. Analg. 1063, 910–915, table of contents. doi: 10.1213/ane.0b013e31816195bc 18292439

[ref48] Landucci E. Pellegrini-Giampietro D. E. Facchinetti F. (2022). Experimental models for testing the efficacy of pharmacological treatments for neonatal hypoxic-ischemic encephalopathy. Biomedicine 10:937. doi: 10.3390/biomedicines10050937, PMID: 35625674 PMC9138693

[ref49] Laviolette S. R. (2007). Dopamine modulation of emotional processing in cortical and subcortical neural circuits: evidence for a final common pathway in schizophrenia? Schizophr. Bull. 33, 971–981. doi: 10.1093/schbul/sbm048, PMID: 17519393 PMC2632330

[ref50] Lee R. M. (1995). Morphology of cerebral arteries. Pharmacol. Ther. 66, 149–173. doi: 10.1016/0163-7258(94)00071-a 7630927

[ref51] Lee J. H. Durand R. Gradinaru V. Zhang F. Goshen I. Kim D. S.. (2010). Global and local fMRI signals driven by neurons defined optogenetically by type and wiring. Nature 465, 788–792. doi: 10.1038/nature09108, PMID: 20473285 PMC3177305

[ref52] Lepore A. C. Fischer I. (2005). Lineage-restricted neural precursors survive, migrate, and differentiate following transplantation into the injured adult spinal cord. Exp. Neurol. 194, 230–242. doi: 10.1016/j.expneurol.2005.02.020, PMID: 15899260

[ref53] Li X. Xiang P. Liang J. Deng Y. Du J. (2022). Global trends and hotspots in esketamine research: a bibliometric analysis of past and estimation of future trends. Drug Des. Devel. Ther. 16, 1131–1142. doi: 10.2147/DDDT.S356284, PMID: 35478936 PMC9037742

[ref54] Liang D. Xia S. Zhang X. Zhang W. (2021). Analysis of brain functional connectivity neural circuits in children with autism based on persistent homology. Front. Hum. Neurosci. 15:745671. doi: 10.3389/fnhum.2021.745671, PMID: 34588970 PMC8473898

[ref55] Lin P. Fang Z. Liu J. Lee J. H. (2016). Optogenetic functional MRI. J. Vis. Exp. 110:53346. doi: 10.3791/53346, PMID: 27167840 PMC4941940

[ref56] Longa E. Z. Weinstein P. R. Carlson S. Cummins R. (1989). Reversible middle cerebral artery occlusion without craniectomy in rats. Stroke 20, 84–91. doi: 10.1161/01.str.20.1.84, PMID: 2643202

[ref57] Luo L. (2021). Architectures of neuronal circuits. Science 373:eabg7285. doi: 10.1126/science.abg7285, PMID: 34516844 PMC8916593

[ref58] McAdams R. M. Fleiss B. Traudt C. Schwendimann L. Snyder J. M. Haynes R. L.. (2017). Long-term neuropathological changes associated with cerebral palsy in a nonhuman primate model of hypoxic-ischemic encephalopathy. Dev. Neurosci. 39, 124–140. doi: 10.1159/000470903, PMID: 28486224 PMC5519434

[ref59] Millar L. J. Shi L. Hoerder-Suabedissen A. Molnár Z. (2017). Neonatal hypoxia ischaemia: mechanisms, models, and therapeutic challenges. Front. Cell. Neurosci. 11:78. doi: 10.3389/fncel.2017.00078, PMID: 28533743 PMC5420571

[ref60] Mirza M. A. Ritzel R. Xu Y. McCullough L. D. Liu F. (2015). Sexually dimorphic outcomes and inflammatory responses in hypoxic-ischemic encephalopathy. J. Neuroinflammation 12:32. doi: 10.1186/s12974-015-0251-6, PMID: 25889641 PMC4359482

[ref61] Nishio Y. Hashimoto M. Ishii K. Ito D. Mugikura S. Takahashi S.. (2014). Multiple thalamo-cortical disconnections in anterior thalamic infarction: implications for thalamic mechanisms of memory and language. Neuropsychologia 53, 264–273. doi: 10.1016/j.neuropsychologia.2013.11.025, PMID: 24321272

[ref62] Northington F. J. Graham E. M. Martin L. J. (2005). Apoptosis in perinatal hypoxic-ischemic brain injury: how important is it and should it be inhibited? Brain Res. Brain Res. Rev. 50, 244–257. doi: 10.1016/j.brainresrev.2005.07.003, PMID: 16216332

[ref63] Oguchi M. Jiasen J. Yoshioka T. W. Tanaka Y. R. Inoue K. Takada M.. (2021). Microendoscopic calcium imaging of the primary visual cortex of behaving macaques. Sci. Rep. 11:17021. doi: 10.1038/s41598-021-96532-z, PMID: 34426639 PMC8382832

[ref64] Olson E. E. McKeon R. J. (2004). Characterization of cellular and neurological damage following unilateral hypoxia/ischemia. J. Neurol. Sci. 227, 7–19. doi: 10.1016/j.jns.2004.07.021, PMID: 15546586

[ref65] Ophelders D. R. Wolfs T. G. Jellema R. K. Zwanenburg A. Andriessen P. Delhaas T.. (2016). Mesenchymal stromal cell-derived extracellular vesicles protect the fetal brain after hypoxia-ischemia. Stem Cells Transl. Med. 5, 754–763. doi: 10.5966/sctm.2015-0197, PMID: 27160705 PMC4878333

[ref66] Ozawa T. Johansen J. P. (2014). Neural circuits: interacting interneurons regulate fear learning. Curr. Biol. 24, R690–R693. doi: 10.1016/j.cub.2014.06.050, PMID: 25093560

[ref67] Pekna M. Pekny M. Nilsson M. (2012). Modulation of neural plasticity as a basis for stroke rehabilitation. Stroke 43, 2819–2828. doi: 10.1161/strokeaha.112.654228, PMID: 22923444

[ref68] Peng C. Liu Y. M. Fei J. F. (2022). Research Progress of neural circuit regeneration. Chinese J. Cell Biol. 4408, 1529–1541.

[ref69] Qiu H. Guo R. Zhang Y. Ying J. Yan Y. Xiong J. (2022). A bibliometric analysis of the hotspots concerning stem cell extracellular vesicles for diabetes in the last 5 years. Front. Public Health 10:868440. doi: 10.3389/fpubh.2022.868440, PMID: 35719682 PMC9201211

[ref70] Rai S. Griffiths K. Breukelaar I. A. Barreiros A. R. Chen W. Boyce P.. (2021). Investigating neural circuits of emotion regulation to distinguish euthymic patients with bipolar disorder and major depressive disorder. Bipolar Disord. 23, 284–294. doi: 10.1111/bdi.13042, PMID: 33369067

[ref71] Rice J. E. 3rd Vannucci R. C. Brierley J. B. (1981). The influence of immaturity on hypoxic-ischemic brain damage in the rat. Ann. Neurol. 9, 131–141. doi: 10.1002/ana.410090206, PMID: 7235629

[ref72] Roohey T. Raju T. N. Moustogiannis A. N. (1997). Animal models for the study of perinatal hypoxic-ischemic encephalopathy: a critical analysis. Early Hum. Dev. 47, 115–146. doi: 10.1016/s0378-3782(96)01773-2, PMID: 9039963

[ref73] Shah A. M. Ishizaka S. Cheng M. Y. Wang E. H. Bautista A. R. Levy S.. (2017). Optogenetic neuronal stimulation of the lateral cerebellar nucleus promotes persistent functional recovery after stroke. Sci. Rep. 7:46612. doi: 10.1038/srep46612, PMID: 28569261 PMC5451884

[ref74] Shi J. Guo H. Liu S. Xue W. Fan F. Fan H.. (2020). Resting-state functional connectivity of neural circuits associated with primary and secondary rewards in patients with bipolar disorder. Soc. Cogn. Affect. Neurosci. 15, 755–763. doi: 10.1093/scan/nsaa100, PMID: 32734286 PMC7511880

[ref75] Shoykhet M. Middleton J. W. (2016). Cardiac arrest-induced global brain hypoxia-ischemia during development affects spontaneous activity organization in rat sensory and motor thalamocortical circuits during adulthood. Front Neural Circuits 10:68. doi: 10.3389/fncir.2016.00068, PMID: 27610077 PMC4996986

[ref76] Smyser C. D. Wheelock M. D. Limbrick D. D. Jr. Neil J. J. (2019). Neonatal brain injury and aberrant connectivity. Neuroimage 185, 609–623. doi: 10.1016/j.neuroimage.2018.07.057, PMID: 30059733 PMC6289815

[ref77] Svoboda J. Litvinec A. Kala D. Pošusta A. Vávrová L. Jiruška P.. (2019). Strain differences in intraluminal thread model of middle cerebral artery occlusion in rats. Physiol. Res. 68, 37–48. doi: 10.33549/physiolres.933958, PMID: 30433803

[ref78] Takatsuru Y. Eto K. Kaneko R. Masuda H. Shimokawa N. Koibuchi N.. (2013). Critical role of the astrocyte for functional remodeling in contralateral hemisphere of somatosensory cortex after stroke. J. Neurosci. 33, 4683–4692. doi: 10.1523/jneurosci.2657-12.2013, PMID: 23486942 PMC6619011

[ref79] Tamura A. Graham D. I. McCulloch J. Teasdale G. M. (1981). Focal cerebral ischaemia in the rat: 1. Description of technique and early neuropathological consequences following middle cerebral artery occlusion. J. Cereb. Blood Flow Metab. 1, 53–60. doi: 10.1038/jcbfm.1981.6, PMID: 7328138

[ref80] Taniguchi H. Andreasson K. (2008). The hypoxic-ischemic encephalopathy model of perinatal ischemia. J. Vis. Exp. 21:955. doi: 10.3791/955, PMID: 19066530 PMC2953967

[ref81] Tennant K. A. Taylor S. L. White E. R. Brown C. E. (2017). Optogenetic rewiring of thalamocortical circuits to restore function in the stroke injured brain. Nat. Commun. 8:15879. doi: 10.1038/ncomms15879, PMID: 28643802 PMC5490053

[ref82] Traudt C. M. McPherson R. J. Bauer L. A. Richards T. L. Burbacher T. M. McAdams R. M.. (2013). Concurrent erythropoietin and hypothermia treatment improve outcomes in a term nonhuman primate model of perinatal asphyxia. Dev. Neurosci. 35, 491–503. doi: 10.1159/000355460, PMID: 24192275 PMC3873854

[ref83] Tuor U. I. Kozlowski P. Del Bigio M. R. Ramjiawan B. Su S. Malisza K.. (1998). Diffusion-and T2-weighted increases in magnetic resonance images of immature brain during hypoxia-ischemia: transient reversal posthypoxia. Exp. Neurol. 150, 321–328. doi: 10.1006/exnr.1997.6766, PMID: 9527902

[ref84] Vahdat S. Pendharkar A. V. Chiang T. Harvey S. Uchino H. Cao Z.. (2021). Brain-wide neural dynamics of poststroke recovery induced by optogenetic stimulation. Sci. Adv. 7:eabd9465. doi: 10.1126/sciadv.abd9465, PMID: 34380610 PMC8357234

[ref85] Vannucci S. J. Hagberg H. (2004). Hypoxia-ischemia in the immature brain. J. Exp. Biol. 207, 3149–3154. doi: 10.1242/jeb.01064 15299036

[ref86] Vannucci R. C. Vannucci S. J. (2005). Perinatal hypoxic-ischemic brain damage: evolution of an animal model. Dev. Neurosci. 27, 81–86. doi: 10.1159/000085978, PMID: 16046840

[ref87] Victor S. Rocha-Ferreira E. Rahim A. Hagberg H. Edwards D. (2022). New possibilities for neuroprotection in neonatal hypoxic-ischemic encephalopathy. Eur. J. Pediatr. 181, 875–887. doi: 10.1007/s00431-021-04320-8, PMID: 34820702 PMC8897336

[ref88] Vitrikas K. Dalton H. Breish D. (2020). Cerebral palsy: an overview. Am. Fam. Physician 1014, 213–220. 32053326

[ref89] Waddell J. Hanscom M. Shalon Edwards N. McKenna M. C. McCarthy M. M. (2016). Sex differences in cell genesis, hippocampal volume and behavioral outcomes in a rat model of neonatal HI. Exp. Neurol. 275, 285–295. doi: 10.1016/j.expneurol.2015.09.003, PMID: 26376217 PMC4688089

[ref90] Wahl A. S. Büchler U. Brändli A. Brattoli B. Musall S. Kasper H.. (2017). Optogenetically stimulating intact rat corticospinal tract post-stroke restores motor control through regionalized functional circuit formation. Nat. Commun. 8:1187. doi: 10.1038/s41467-017-01090-6, PMID: 29084962 PMC5662731

[ref91] Wang X. Yin Z. Sun Q. Jiang X. Chao L. Dai X.. (2021). Comparative study on the functional connectivity of amygdala and hippocampal neural circuits in patients with first-episode schizophrenia and other high-risk populations. Front. Psych. 12:627198. doi: 10.3389/fpsyt.2021.627198, PMID: 34539456 PMC8442955

[ref92] Ward N. S. Cohen L. G. (2004). Mechanisms underlying recovery of motor function after stroke. Arch. Neurol. 61, 1844–1848. doi: 10.1001/archneur.61.12.1844, PMID: 15596603 PMC3713312

[ref93] Wassink G. Gunn E. R. Drury P. P. Bennet L. Gunn A. J. (2014). The mechanisms and treatment of asphyxial encephalopathy. Front. Neurosci. 8:40. doi: 10.3389/fnins.2014.00040, PMID: 24578682 PMC3936504

[ref94] Wen L. Sun J. Chen X. Du R. (2020). miR-135b-dependent downregulation of S100B promotes neural stem cell differentiation in a hypoxia/ischemia-induced cerebral palsy rat model. Am. J. Physiol. Cell Physiol. 319, C955–C966. doi: 10.1152/ajpcell.00481.2019, PMID: 32491925

[ref95] Widerøe M. Brekken C. Kavelaars A. Pedersen T. B. Goa P. E. Heijnen C.. (2011). Longitudinal manganese-enhanced magnetic resonance imaging of delayed brain damage after hypoxic-ischemic injury in the neonatal rat. Neonatology 100, 363–372. doi: 10.1159/000328705, PMID: 21791927

[ref96] Winnubst J. Bas E. Ferreira T. A. Wu Z. Economo M. N. Edson P.. (2019). Reconstruction of 1,000 projection neurons reveals new cell types and organization of long-range connectivity in the mouse brain. Cells 179, 268–281.e13. doi: 10.1016/j.cell.2019.07.042, PMID: 31495573 PMC6754285

[ref97] Yager J. Y. Ashwal S. (2009). Animal models of perinatal hypoxic-ischemic brain damage. Pediatr. Neurol. 40, 156–167. doi: 10.1016/j.pediatrneurol.2008.10.025 19218028

[ref98] Yamamoto N. Marks W. D. Kitamura T. (2021). Cell-type-specific optogenetic techniques reveal neural circuits crucial for episodic memories. Adv. Exp. Med. Biol. 1293, 429–447. doi: 10.1007/978-981-15-8763-4_28, PMID: 33398831 PMC8612024

[ref99] Yan S. Li Y. Lu J. Tian T. Zhang G. Zhou Y.. (2022). Structural and functional alterations within the Papez circuit in subacute stroke patients. Brain Imaging Behav. 16, 2681–2689. doi: 10.1007/s11682-022-00727-5, PMID: 36222964

[ref100] Yang H. Shan W. Fan J. Deng J. Luan G. Wang Q.. (2022). Mapping the neural circuits responding to deep brain stimulation of the anterior nucleus of the thalamus in the rat brain. Epilepsy Res. 187:107027. doi: 10.1016/j.eplepsyres.2022.107027, PMID: 36201994

[ref101] Zhao Y. Zhang Z. Qin S. Fan W. Li W. Liu J.. (2021). Acupuncture for Parkinson's disease: efficacy evaluation and mechanisms in the dopaminergic neural circuit. Neural Plast. 2021:9926445. doi: 10.1155/2021/9926445, PMID: 34221005 PMC8221898

[ref102] Zhu H. Li Q. Feng M. Chen Y. X. Li H. Sun J. J.. (2011). A new cerebral hemorrhage model in cynomolgus macaques created by injection of autologous anticoagulated blood into the brain. J. Clin. Neurosci. 18, 955–960. doi: 10.1016/j.jocn.2010.11.038, PMID: 21601461

[ref103] Zwemer C. F. Whitesall S. E. D'Alecy L. G. (1995). Hypoxic cardiopulmonary-cerebral resuscitation fails to improve neurological outcome following cardiac arrest in dogs. Resuscitation 29, 225–236. doi: 10.1016/0300-9572(94)00848-a, PMID: 7667554

